# Intron-derived small RNAs for silencing viral RNAs in mosquito cells

**DOI:** 10.1371/journal.pntd.0010548

**Published:** 2022-06-23

**Authors:** Priscilla Y. L. Tng, Leonela Z. Carabajal Paladino, Michelle A. E. Anderson, Zach N. Adelman, Rennos Fragkoudis, Rob Noad, Luke Alphey

**Affiliations:** 1 Arthropod Genetics Group, The Pirbright Institute, Pirbright, United Kingdom; 2 Pathobiology and Population Sciences, The Royal Veterinary College, Hatfield, United Kingdom; 3 Department of Entomology, Texas A&M University, College Station, Texas, United States of America; 4 Arbovirus Pathogenesis Group, The Pirbright Institute, Pirbright, United Kingdom; Connecticut Agricultural Experiment Station, UNITED STATES

## Abstract

*Aedes aegypti* and *Ae*. *albopictus* are the main vectors of mosquito-borne viruses of medical and veterinary significance. Many of these viruses have RNA genomes. Exogenously provided, e.g. transgene encoded, small RNAs could be used to inhibit virus replication, breaking the transmission cycle. We tested, in *Ae*. *aegypti* and *Ae*. *albopictus* cell lines, reporter-based strategies for assessing the ability of two types of small RNAs to inhibit a chikungunya virus (CHIKV) derived target. Both types of small RNAs use a *Drosophila melanogaster* pre-*miRNA-1* based hairpin for their expression, either with perfect base-pairing in the stem region (shRNA-like) or containing two mismatches (miRNA-like). The pre-*miRNA-1* stem loop structure was encoded within an intron; this allows co-expression of one or more proteins, e.g. a fluorescent protein marker tracking the temporal and spatial expression of the small RNAs *in vivo*. Three reporter-based systems were used to assess the relative silencing efficiency of ten shRNA-like siRNAs and corresponding miRNA-like designs. Two systems used a luciferase reporter RNA with CHIKV RNA inserted either in the coding sequence or within the 3’ UTR. A third reporter used a CHIKV derived split replication system. All three reporters demonstrated that while silencing could be achieved with both miRNA-like and shRNA-like designs, the latter were substantially more effective. Dcr-2 was required for the shRNA-like siRNAs as demonstrated by loss of inhibition of the reporters in Dcr-2 deficient cell lines. These positive results in cell culture are encouraging for the potential use of this pre-*miRNA-1*-based system in transgenic mosquitoes.

## Author summary

Mosquitoes are important globally, spreading viral diseases worldwide. Chikungunya virus causes epidemics of disease in people. Here we have investigated using two types of small RNAs and pathways inherent in *Aedes aegypti* mosquitoes to target a piece of the chikungunya virus’s genome, potentially preventing viral replication. We express these small RNAs using a pre-*miRNA-1* based system, inserted into the intron within a commonly used promoter. We have used reporter systems in cell lines which can give preliminary indications of how these systems might work in mosquitoes. Our results indicate that short-hairpin-like designs are more effective than micro-RNA-like designs at knocking down expression of their targets. This knock-down requires Dcr-2 indicating that the short-hairpin-like RNAs are likely using the endo-siRNA pathway to degrade mRNA which contains their complementary RNA.

## Introduction

*Aedes aegypti* and *Ae*. *albopictus* are major vectors of arboviruses such as chikungunya virus (CHIKV), which causes major epidemics of disease in people [1]. Although several vaccine options against CHIKV are in development [2], disease control for this and most other arboviruses relies heavily on vector control. Many antiviral strategies and preliminary laboratory-based models have been developed in *Ae*. *aegypti* [3–6] but none have been explored or implemented in *Ae*. *albopictus*, although the implementation of control strategies against *Ae*. *aegypti* alone may be insufficient to break disease transmission, which may in some cases be maintained by *Ae*. *albopictus*.

The development of a mosquito population replacement system with genes conferring resistance to virus infection or transmission provides attractive alternatives or complements to population suppression strategies. Small RNAs can be effective against viruses *in vivo* in mosquitoes [5–7]. However, for this strategy to be successful it requires both suitable levels of expression, and expression of the small RNAs in the right tissues to ensure contact with the virus. Monitoring temporal and tissue specific expression of short interfering RNAs (siRNAs) in live transgenic animals is challenging. One possible solution to this problem is to insert siRNA sequences in the intron of a gene expressing a fluorescent reporter. Expression of the reporter in the animal then indicates gene expression.

In *Ae*. *albopictus*, miRNAs have been successfully expressed *in vitro* and *in vivo* from synthetic intronic sequences in a densoviral delivery system derived from natural mosquito specific viruses [8]. This design included a fluorescent protein reporter expressed only if the miRNA expressing intron was correctly processed and spliced. Liu et al. also established that the synthetic miRNAs could also modulate the expression of targeted endogenous miRNAs and downregulate targeted genes.

Using the *Drosophila melanogaster* pre-*miRNA-1* stem loop sequence, Haley et al. [9] generated transgenic *D*. *melanogaster* strains expressing effective miRNA arrays targeting endogenous genes modulating wing development. These authors also demonstrated that synthetic targets in the 3’ UTR of a reporter could be suppressed by the miRNA arrays *in vitro*.

Here we report use of a *D*. *melanogaster* pre-*miRNA-1* stem loop structure within an intron downstream of the constitutively expressed *Ae*. *aegypti polyubiquitin* gene (*PUb*) promoter [10]. This allowed co-expression of the small RNAs with luciferase or fluorophore-based reporters from the same transcript. Using this system, we successfully expressed a panel of synthetic small RNAs targeting CHIKV RNA and demonstrated downregulation of reporters containing the target sequence. We tested these constructs in *Ae*. *aegypti* and *Ae*. *albopictus* cells, validating the system and identifying short RNAs that efficiently silence these reporters in cells from both mosquito species. Interestingly, a comparison of perfectly complementary small RNAs (shRNA-like) and variants which contained two mismatches to mimic the structure of the endogenous *D*. *melanogaster* pre-*miRNA-1* (miRNA-like) showed that the shRNA-like-based designs were better at suppressing targets. Furthermore, activity of synthetic small RNAs that are fully complementary (shRNA-like) was substantially dependent on Dcr-2 in mosquito cells, indicating that processing primarily occurred through the Dcr-2 dependent endogenous siRNA pathway, rather than the miRNA pathway, which primarily uses Dcr-1. We also showed the advantage of synthetic viral split-replication systems, and chimeric reporters, for analyzing the efficacy of small RNAs against viruses and other targets of choice.

## Methods

### Small RNA design

**Fig 1:**
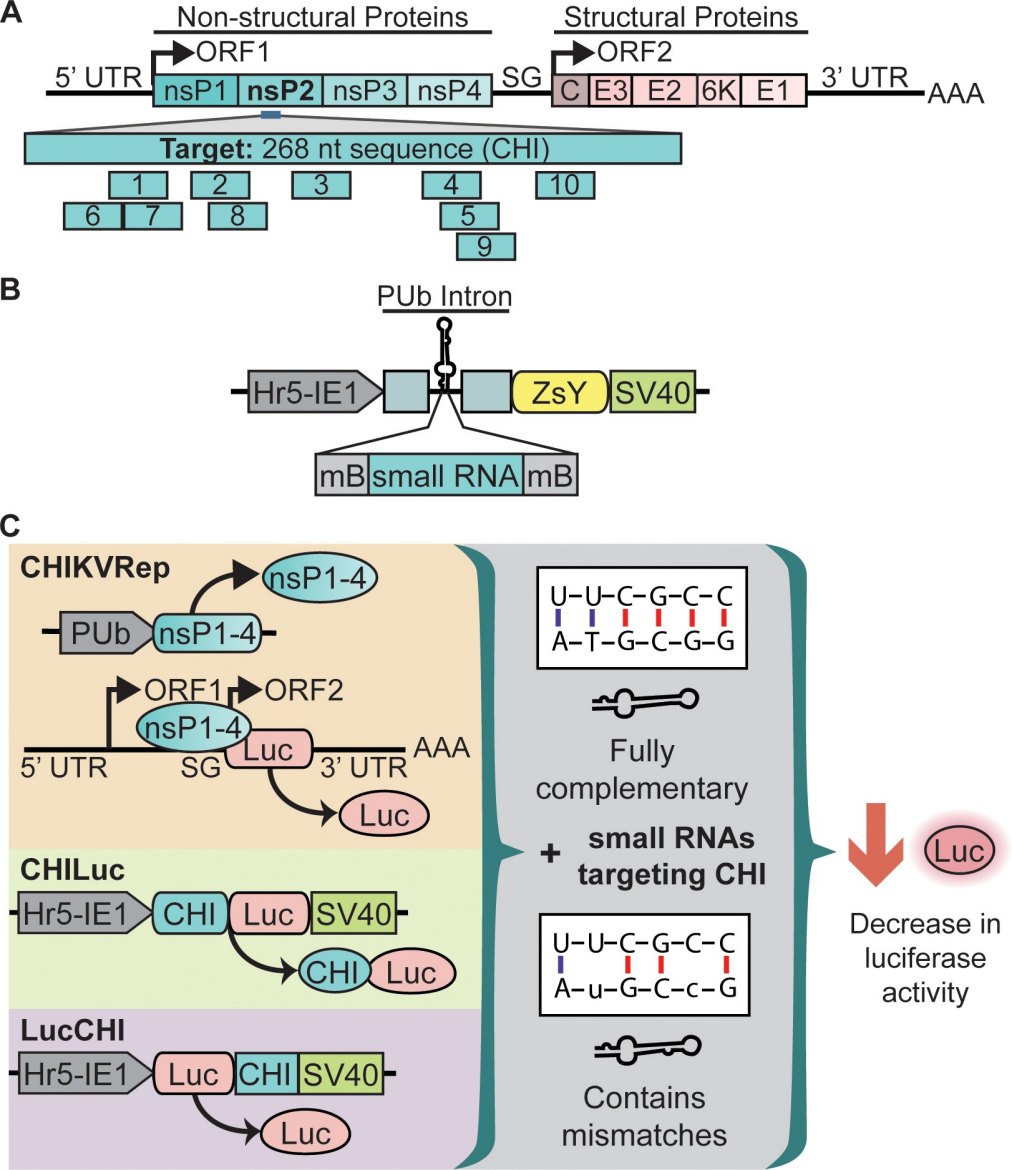
Experimental designs. **(A)** Schematic of the CHIKV genome and the location of the 268 nt nsP2 target region (CHI) and each of the 10 sites targeted by small RNAs. **(B)** Schematic of small RNA expressing plasmids. **(C)** Strategy for testing small RNA mediated suppression of a CHIKV split replication system (CHIKVRep, replication complex nsP1-4 and viral reporter), chimeric firefly luciferase reporter (CHILuc) and synthetic reporter with the target sequence in the 3’ UTR (LucCHI). Small RNAs containing sequence mismatches (nt 2 and 11) or fully complementary sequences were assessed for their ability to target viral sequences in mosquito cell lines. AAA: polyadenylation signal, Hr5-IE1: *Autographa californica* nuclear polyhedrosis virus homologous region 5 enhancer-immediate-early gene 1 promoter, Luc: luciferase, mB: *Drosophila melanogaster* pre-*miRNA-1* miRNA backbone, nt: nucleotide, ORF: open reading frame, PUb intron: *Ae*. *aegypti polyubiquitin* gene intron, SG: CHIKV subgenomic promoter, SV40: simian virus 40 polyadenylation signal, UTR: untranslated regions, ZsY: ZsYellow fluorescent protein.

The *D*. *melanogaster* pre-*miRNA1* stem loop sequence validated by Haley et al. [9] was modified to target a 268 nt conserved region of the nsP2 viral protease coding region of CHIKV (CHI, Fig 1A). The conserved region was identified by aligning sequences from different CHIKV isolates deposited in NCBI between 2006 and 2019 (La Reunion 2006: DQ443544, Congo 2019: MK935343, China 2019: MN402889, Myanmar 2019: MN402887, Thailand 2018: MK468801, Italy 2017: MK120196, Bangladesh 2017: MK468610, Sudan 2018: MK163628). Pre-*miRNA-1* was inserted into the intron of the *PUb* promoter sequence (AAEL003888-RB) to allow the co-expression of a reporter (Fig 1B). The siDESIGN Center (Horizon Discovery Group plc) was used to identify potential target sites. The output list was reduced to ten sites that showed minimal homology to the RNA transcripts of *Ae*. *aegypti* (AaegL3 and AaegL5), to reduce interaction with endogenous sequences, and independently of their location within the target [11,12] (S1 Fig, and S1 Table). To mimic the structure of pre-*miRNA-1* (S2A Fig) in the synthetic miRNAs, nucleotide mismatches were designed into the sequence at nucleotides 2 and 11 within the sense strand (S2B and S2D Fig), whilst the sense strand was designed to be fully complementary to the antisense strand in shRNA-like siRNAs (S2C and S2E Fig).

### Plasmid construction

The AGG1239:pmiR1-ZsY vector (Genbank accession no. MZ514802, Fig 1B) used for the expression of the small RNAs was constructed as follows: plasmid AGG1186:pHr5Fluc [13] was linearized with *Sal*I and *Nhe*I. The *Autographa californica* nuclear polyhedrosis virus homologous region 5 enhancer-immediate-early gene 1 (Hr5-IE1) promoter [14,15] and SV40 3’ UTR were reformed, and *Asc*I and *Nco*I restriction sites and the ZsYellow (ZsY) marker protein (from plasmid AGG1328:pCas13b, Genbank accession no. MT119959.1) were cloned into the vector in a four-way ligation to form the intermediate vector AGG1239Int (Genbank accession no. MZ514801). Halves of the pre-*miRNA-1* backbone were added to fragments of the *Ae*. *aegypti PUb* intron by PCR and the intron was reformed in a three-way ligation with the AGG1239Int vector, which was linearized with *Asc*I and *Nco*I. *Hind*III and *Bam*HI restriction sites were designed into the pre-*miRNA-1* backbone for the insertion of miRNA and shRNA oligos.

A panel of small RNAs was designed (S1 Table). The forward and reverse strand of each molecule was synthesized (Sigma-Aldrich) and annealed to form double stranded DNA fragments for insertion into the linearized AGG1239:pmiR1-ZsY vector (S1 Table). An AmCyan targeting miRNA-like or shRNA-like siRNA (miRNT and shRNT, S1 Table) was designed as the negative control for all the experiments and cloned into the same vector. A miRNA-like and shRNA-like siRNA against firefly luciferase (FL) (miRT and shRT, S1 Table) was used as positive control when the CHILuc and LucCHI reporters were tested. All inserts were confirmed with Sanger sequencing and folding of the pre-*miRNA-1* stem loop was modeled with MFold [16].

For the CHIKVRep split-replication system we used plasmids Ubi-P1234-CHIKV (pCHIKVRep2) and AGG1521:pCHIKVRep1 [13,17–19]. Plasmid Ubi-P1234-CHIKV (pCHIKVRep2) provides the replicase complex, while plasmid AGG1521:pCHIKVRep1 contains the subgenomic promoter, and nanoluciferase (NL) replacing the structural proteins. Both plasmids need to be present in the cell for the expression of the fluorophore. Details on the mechanism of action of this reporter are included in S4 Fig. The CHILuc reporter was plasmid AGG1221:CHIKVLuc previously described in Tng et al. [13]. The LucCHI reporter (plasmid AGG1220:pLucCHIKV, Genbank accession no. MZ514803) was generated by digesting AGG1186:pHr5Fluc with *Fse*I and *Xba*I, and the 268 bp fragment of the nsP2 sequence of CHIKV LR2006-OPY1 strain (ECSA genotype) was inserted into the 3’ UTR of FL.

AGG1080:pRL-Opie2 *Renilla* luciferase (RL) [10] or AGG1186:pHr5Fluc FL [13] plasmids were used as reference to normalize data for transfection efficiency where appropriate.

### Cells

Aag2 (RRID:CVCL_Z617), AF05 (RRID:CVCL_A8M5) and AF319 (RRID:CVCL_A8M6) cells (*Ae*. *aegypti* derived), U4.4 (RRID:CVCL_Z820) and C6/36 (RRID:CVCL_Z230) cells (*Ae*. *albopictus* derived) were used for transfections. AF319 cells are Dcr-2 CRISPR knockout from the AF05 single cell derived parental cell line [20–22], while C6/36 cells have a non-functional Dcr-2 [23–25]. All mosquito cell lines were grown in Leibovitz-15 media (Gibco) supplemented with 10% fetal bovine serum (Labtech), 100 I.U./ml penicillin with 100μg/ml streptomycin (Gibco), and 10% tryptose phosphate broth (Gibco) at 28°C without CO2 or humidity control.

### Cell transfections

Cell transfections were carried out as previously described [13]. For each experiment, three independent transfections with at least five replicates were performed. AGG1080:pRL-Opie2 or AGG1186:pHr5FLuc (see above) were used as transfection control where applicable. Amounts of plasmid and RNA mixtures used for transfections were optimized such that the total mass did not exceed 250 ng. A shRNA-like and miRNA-like siRNA targeting AmCyan, a gene not present in the reporter RNA, were used as a control to test the specificity of the effect in all the experiments. A shRNA-like and miRNA-like siRNA targeting FL was used as positive control when the LucCHI and CHILuc reporters were tested. Specific details for transfection mixtures are listed in S17 Table.

### Luciferase assays

Luciferase assays for transfections with the CHIKVRep split replication system, CHILuc and LucCHI were performed as previously described [13,26] with the Dual-Luciferase Reporter Assay (Promega) on a GloMax multi+ plate reader (Promega) (S4 and S5 Figs). The assays were carried out according to manufacturer’s instructions.

### Statistical analysis

In the experiments with the CHIKVRep split replication system (S4 Fig), the NL activity was normalized against FL activity (NL/FL) to correct for transfection efficiency in each well. In experiments using CHILuc or LucCHI synthetic reporters (S5 Fig), FL activity was normalized against RL activity (FL/RL). The NL/FL or FL/RL ratios were analyzed with R (version 4.0.2). Linear mixed effect models fit by restricted maximum likelihood were preferentially used to investigate the effect of the small RNAs tested on the expression levels of the reporters used. The lmer function in the lme4 package [27] and the lmerTest package [28] were used to calculate the t-tests using Sattherthwaite approximations to degrees of freedom. Within each experiment, data were transformed as appropriate to fit a normal distribution and models were run separately for each cell line and small RNA, where applicable. The small RNAs used were set as the categorical fixed factor and the experiment as a random factor. Diagnostic plots of residuals were checked to ensure that there was constant variance between residuals and model assumptions were met. One-way ANOVA was used where a model could not be fitted and Kruskal Wallis test was used where data could not be transformed to normality. Tukey’s honest significant differences test (multcomp package [29]) or Dunn’s test (FSA package [30]) were used for post-hoc analyses where applicable.

## Results

### shRNA-like siRNAs were more effective than miRNA-like siRNAs in targeting a CHIKV split replication system and viral reporters

We investigated differences in function between synthetic small RNAs that possess sequence mismatches that mimicked the previously validated *D*. *melanogaster* pre-*miRNA-1* [9], and small RNAs with the same backbone but with fully complementary sequences. Sequence mismatches are thought to be crucial in miRNA identification and processing, including strand-specific loading of processed miRNAs into the miRISC complex [31]. We designed a panel of ten small RNAs against a 268 nt conserved region of nsP2 of CHIKV virus (CHI, Fig 1A and S1 Table). Targets were selected using siDESIGN Center, a small interfering RNA design software, with the assumption that processed small RNAs would have similar targeting properties as siRNAs (S1 Fig). Small RNAs with mismatches at nucleotides 2 and 11 (miRNA-like) or fully complementary (shRNA-like) were designed for each target (S2 Fig and S1 Table). These were expressed from a *D*. *melanogaster* pre-*miRNA-1* miRNA backbone structure within an intron derived from the *Ae*. *aegypti PUb* gene [10] downstream from the broadly-active Hr5-IE1 promoter [14] (Fig 1B). This intronic expression design enabled the co-expression of the reporter fluorophore ZsY for visualization purposes (Fig 1B). ZsY expression was confirmed in *Ae*. *aegypti* and *Ae*. *albopictus* derived cells using selected small RNA constructs (S3 Fig). The ability of each type of small RNAs to mediate silencing was analyzed in *Ae*. *aegypti* derived Aag2 and *Ae*. *albopictus* derived U4.4 cells. Since CHIKV is a significant pathogen (UK: ACDP3), these assessments were performed with (1) a split replication system “CHIKVRep” [13,17,18] (S4 Fig), (2) a chimeric luciferase reporter “CHILuc” with the targeted viral sequence in the 5’ region of the coding region of FL [13], and (3) a synthetic luciferase reporter “LucCHI” with targeted viral sequence in the 3’ UTR (Fig 1C), in a dual-luciferase assay (S4 and S5 Figs). Compared with virus-based assays, the split replication system separates expression of the replicase from the reporter readout; inhibition of replicase may reduce the amount of replication and hence the amount of luciferase other than by direct inhibition. This should make reporter response more linearly related to replicase inhibition, though it may also lead to an underestimate of the effect on virus replication. It also allows relatively high-throughput analysis of different siRNAs or other replicase-targeting effectors, in a low-risk (pathogen-free) context. A non-targeting small RNA (shRNT and miRNT, S1 Table) was used as negative control for all three reporters, and shRNA-like and miRNA-like targeting FL (shRT and miRT, S1 Table) were used as positive controls when CHILuc and LucCHI were tested.

**Fig 2:**
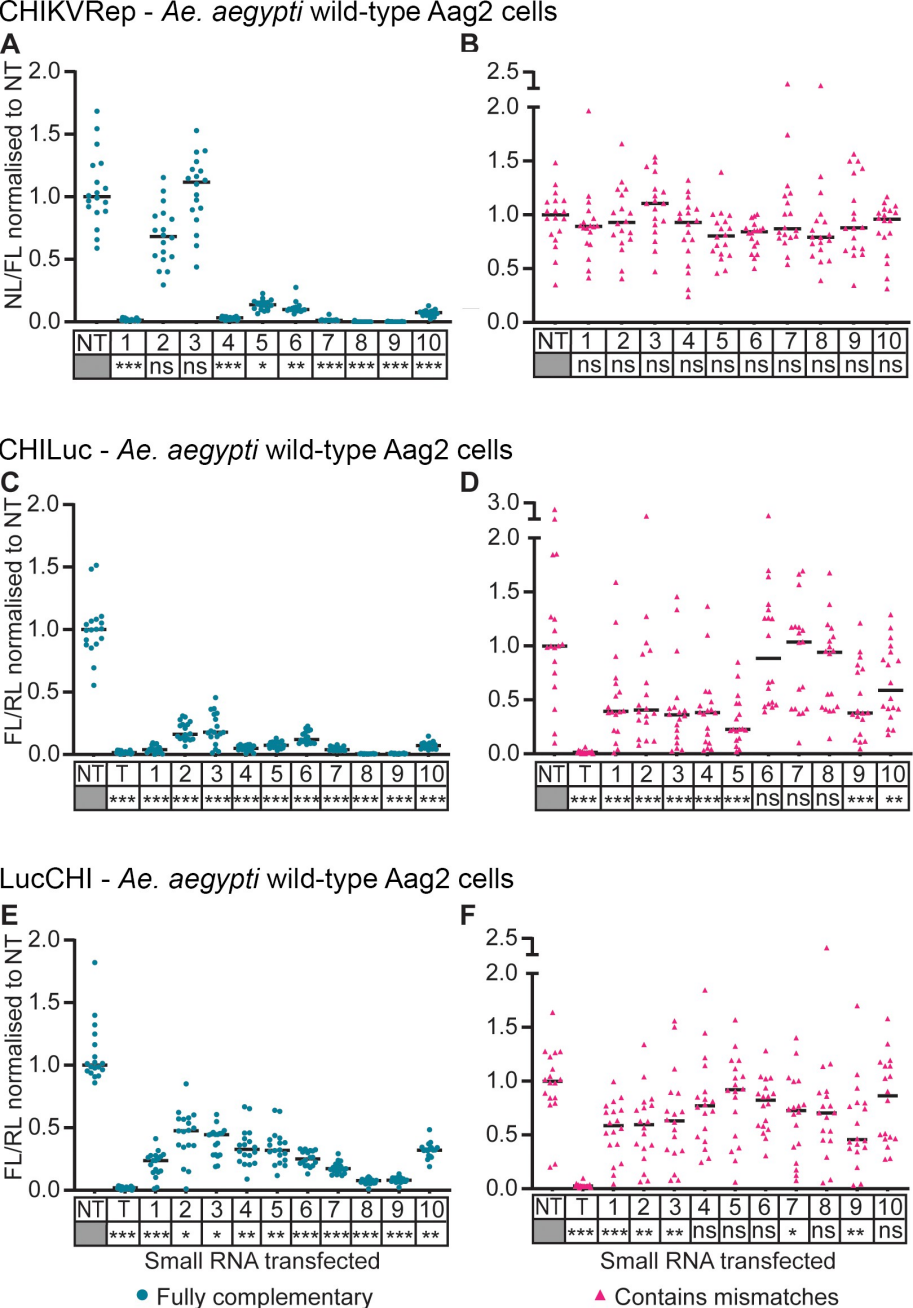
Effect of small RNAs on expression of a CHIKV split replication system and different reporter systems containing viral sequences in *Ae*. *aegypti* wild-type Aag2 cells. Plasmids expressing selected shRNA-like small RNAs (Fully complementary, blue) or miRNA-like small RNAs (Contains mismatches, pink) were co-transfected with a split replication system with nanoluciferase (NL) under the control of a CHIKV subgenomic promoter, and a plasmid expressing firefly luciferase (FL) as control for transfection efficiency (**A, B**); a chimeric CHILuc reporter containing the target sequence within the protein coding region (**C, D**); or a LucCHI reporter which has the target sequence in the 3’ UTR (**E, F**). A plasmid expressing *Renilla* luciferase (RL) as control for transfection efficiency was used in C-F, as well as small RNA targeting FL as positive control (T). Numbers 1 to 10 represent each small RNA tested. Each line represents the median values of NL and FL ratios (NL/FL) or FL and RL ratios (FL/RL) normalized to the non-targeting control (NT). Three independent transfections with at least five replicates each. Each data point represents a replicate. (**B, C, D**) Linear mixed effect model, (**A, E, F**) Kruskal Wallis. Statistical significance of the silencing effect between each small RNA and the NT is denoted under each small RNA name (*** P<0.001, ** P<0.01, * P<0.05, ns: not significant) (S2–S7 Tables).

The silencing efficiency of the shRNA-like and miRNA-like siRNAs was tested in Aag2 cells (Figs 2 and S4 and S5). Eight of ten shRNA-like siRNAs demonstrated strong suppression ability against CHIKVRep, indicated by a significant reduction in the expression of NL compared to the non-targeting control (NT) (P < 0.05) (Fig 2A). In contrast, the miRNA-like designs were unable to suppress the expression of CHIKVRep, as NL expression did not statistically differ from that of the cells transfected with NT (P > 0.05) (Fig 2B). These effects were confirmed using CHILuc, with the CHIKV target sequence within the coding region of the mRNA (Fig 2C and 2D). The strong CHILuc suppression obtained with shRNA-like siRNAs was similar to that observed with CHIKVRep, except that strong suppression was observed with all ten shRNA-like siRNAs (Fig 2C). Unlike with CHIKVRep, statistically significant suppression of CHILuc was observed with some miRNA-like siRNAs using the luciferase reporter RNA (P < 0.05) (Fig 2D miRNAs 1 to 5, 9 and 10), though weaker than for shRNA-like siRNAs. To extend these results, the effect of moving the CHIKV target sequence to the 3’ UTR of the target RNA (LucCHI) was investigated (Fig 2E and 2F, respectively). Although all shRNA-like siRNAs were effective with this target (P < 0.05), the degree of silencing was less than with CHILuc, and only five miRNA-like siRNAs were effective compared to seven in CHILuc and none in CHIKVRep.

**Fig 3:**
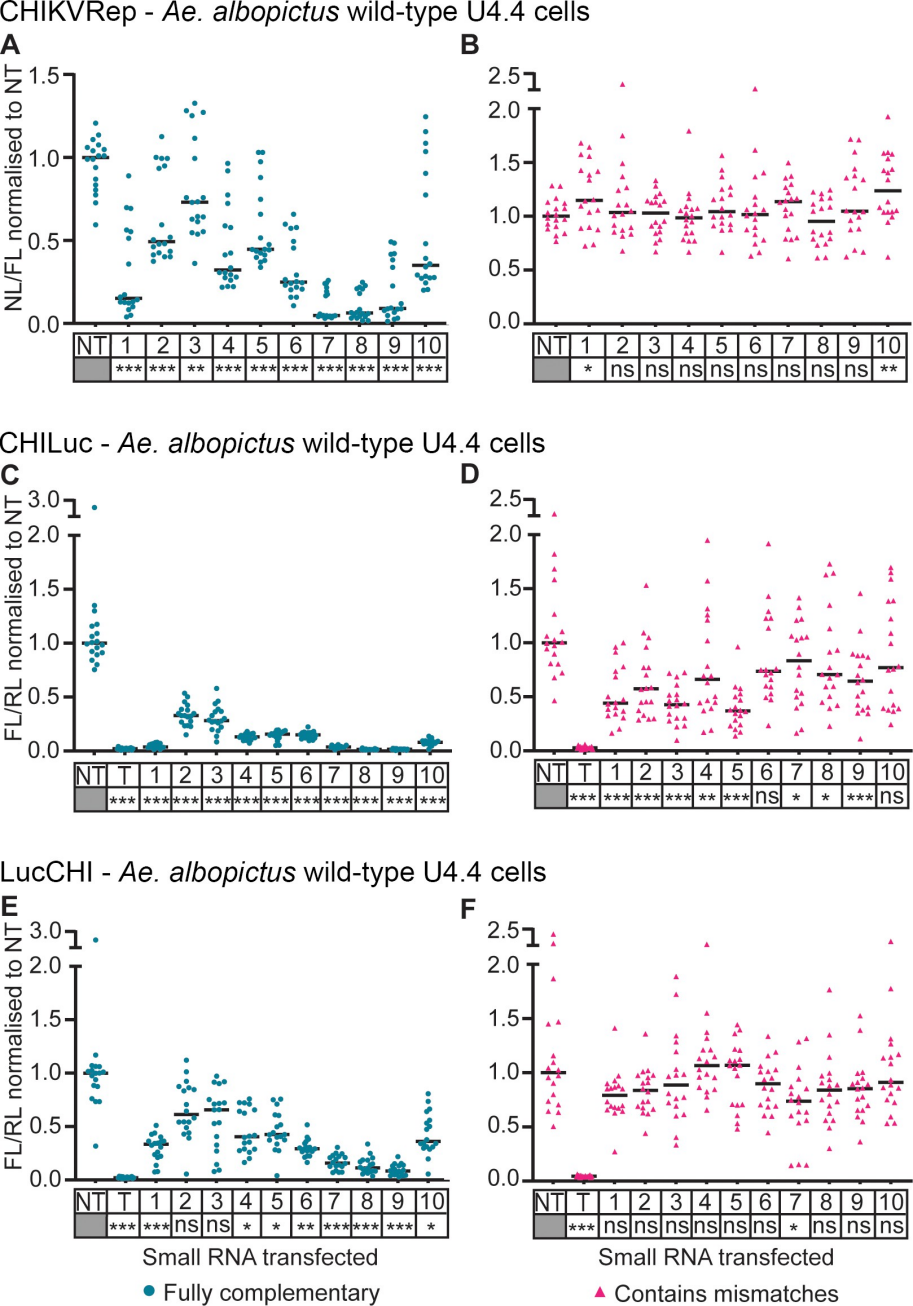
Effect of small RNAs on expression of a CHIKV split replication system and different reporter systems containing viral sequences in *Ae*. *albopictus* wild-type U4.4 cells. Plasmids expressing selected shRNA-like small RNAs (Fully complementary, blue) or miRNA-like small RNAs (Contains mismatches, pink) were co-transfected with a split replication system with nanoluciferase (NL) under the control of a CHIKV subgenomic promoter, and a plasmid expressing firefly luciferase (FL) as control for transfection efficiency (**A, B**), a chimeric CHILuc reporter containing the target sequence within the protein coding region (**C, D**), or a LucCHI reporter which has the target sequence in the 3’ UTR (**E, F**). A plasmid expressing *Renilla* luciferase (RL) as control for transfection efficiency was used in C-F, as well as small RNA targeting FL as positive control (T). Numbers 1 to 10 represent each small RNA tested. Each line represents the median values of NL and FL ratios (NL/FL) or FL and RL ratios (FL/RL) normalized to the non-targeting control (NT). Three independent transfections with at least five replicates each. Each data point represents a replicate. (**A-D**) Linear mixed effect model, (**E, F**) Kruskal Wallis. Statistical significance of the silencing effect between each small RNA and the NT is denoted under each small RNA name (*** P<0.001, ** P<0.01, * P<0.05, ns: not significant) (S8–S13 Tables).

Similar results were observed when the panel of ten small RNAs was screened using *Ae*. *albopictus* derived wild-type U4.4 cells (Fig 3). As with Aag2 cells, all the shRNA-like siRNAs were more effective in suppressing CHIKVRep expression (Fig 3A) compared to miRNA-like siRNAs (P < 0.05) (Fig 3B), although suppression was weaker (compare Figs 2A and 3A). Suppression was generally more effective with CHILuc and LucCHI (Fig 3C, 3D, 3E and 3F, respectively), than with CHIKVRep (Fig 3A and 3B). With all tested shRNA-like siRNAs, knockdown of CHILuc (Fig 3C) was higher in comparison to LucCHI (Fig 3E). Interference with miRNA-like siRNAs was observed with CHILuc (Fig 3D) and LucCHI (Fig 3F), but levels were not as strong as the positive control (T) and were generally weaker than the corresponding shRNA-like siRNAs.

These results indicate that, in our design, fully-complementary siRNAs silenced the CHIKV-derived targets more efficiently. The lower silencing observed with shRNA-like siRNAs 2 and 3 may reflect target availability, e.g. due to folding and/or protein association, and highlights the importance of testing several potential siRNAs, as they can clearly vary in their silencing efficiency.

### Effective suppression of expression by shRNA-like siRNAs requires Dcr-2

Previous studies in *D*. *melanogaster* reported that hairpin structures with a high degree of complementarity tend to be sorted into the Dcr-2/Ago2 pathway [32–34]. Since the synthetic shRNA-like siRNAs were designed to be fully complementary, we investigated whether the strong suppression observed with shRNA-like siRNAs was associated with differential sorting of these molecules, potentially into the Dcr-2 pathway, which is mainly responsible for siRNA-mediated defense against viral infections in mosquitoes [35–37]. To assess this, we used Dcr-2 knockout *Ae*. *aegypti* AF319 or Dcr-2 deficient *Ae*. *albopictus* C6-36 cell lines with four highly-effective small RNAs from the panel above with least homology to the *Ae*. *aegypti* transcriptome (shRNA-like and miRNA-like 6, 7, 8 and 9). AF05 cells, a clonal derivative of Aag2 and the Dcr-2-containing cell line from which the AF319 Dcr-2 knockout cell line was derived, were used as a Dcr-2^+^ control.

**Fig 4:**
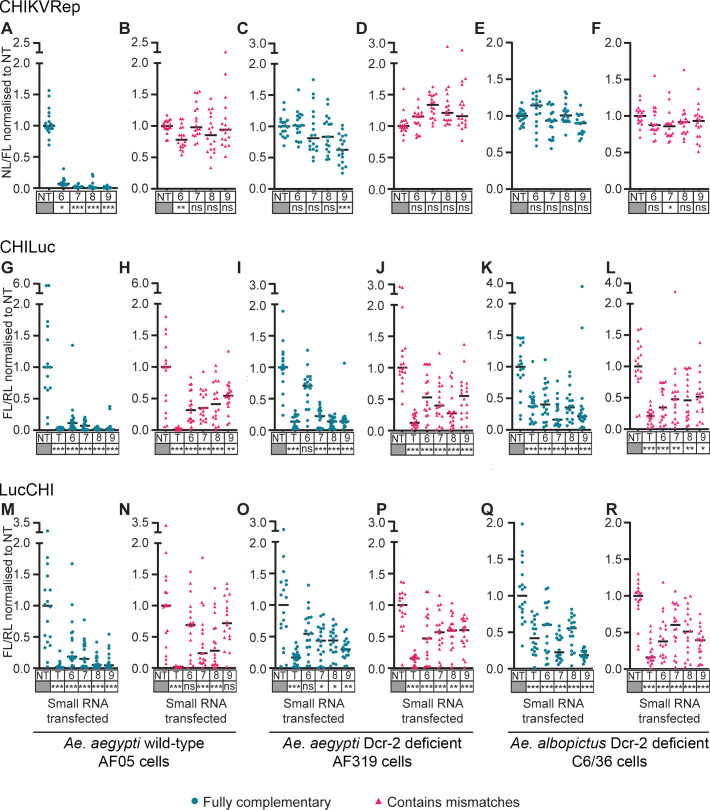
**Effect of selected small RNAs on expression of a CHIKV split replication system and different reporter systems containing viral sequences in *Ae*. *aegypti* AF05 (A, B, G, H, M, N) cells, and Dcr-2 deficient *Ae*. *aegypti* AF319 (C, D, I, J, O, P) and *Ae*. *albopictus* C6/36 (E, F, K, L, Q, R) cells**. Plasmids expressing selected shRNA-like small RNAs (Fully complementary, blue) or miRNA-like small RNAs (Contains mismatches, pink) were co-transfected with a split replication system with nanoluciferase (NL) under the control of a CHIKV subgenomic promoter, and a plasmid expressing firefly luciferase (FL) as control for transfection efficiency (**A-F**), a chimeric CHILuc reporter containing the target sequence within the protein coding region (**G-L**), or a LucCHI reporter which has the target sequence in the 3’ UTR (**M-R**). A plasmid expressing *Renilla* luciferase (RL) as control for transfection efficiency was used in G-R, as well as small RNA targeting FL as positive control (T). Numbers 6 to 9 represent each small RNA tested. Each line represents the median values of NL and FL ratios (NL/FL) or FL and RL ratios (FL/RL) normalized to the non-targeting control (NT). Three independent transfections with at least five replicates each, and each data point represents a replicate. (**B, C, E, F, G, H, J, K, N, P-R**) Linear mixed effect model, (**A, D, I, L, O**) Kruskal Wallis, (**M**) one-way ANOVA. Statistical differences indicate higher silencing efficiency of the siRNA compared to the non-targeting siRNA control. Statistical significance of the silencing effect between each small RNA and the NT is denoted under each small RNA name (*** P<0.001, ** P<0.01, * P<0.05, ns: not significant) (S14–S16 Tables).

Similar to Aag2 cells, strong suppression of CHIKVRep was observed with shRNA-like siRNAs 6–9 in AF05 cells (P < 0.05) (Fig 4A) and little to no silencing with the corresponding miRNA-like siRNAs (P > 0.05) (Fig 4B). In contrast, little to no suppression was induced by shRNA-like siRNAs in Dcr-2 deficient AF319 cells (Fig 4C) and C6/36 cells (Fig 4E) (P > 0.05), and these levels were similar to those of the matching miRNA-like siRNAs (Fig 4D and 4F). Likewise, shRNA-like siRNAs strongly suppressed CHILuc and LucCHI in AF05 cells (P < 0.05) (Fig 4G and 4M), with weaker silencing with miRNA-like siRNAs (Fig 4H and 4N). Silencing ability of the same shRNA-like siRNAs was somewhat reduced in the absence of Dcr-2 in AF319 (Fig 4I and 4O) and C6/36 cells (Fig 4K and 4Q). Weak suppression of both viral reporters was observed with miRNA-like siRNAs (Fig 4J and 4L and 4P and 4R), similar to the levels observed in Aag2 (Fig 2D and 2F), AF05 (Fig 4H and 4N) and U4.4 (Fig 3D and 3F) cells.

These results indicate that fully complementary shRNA-like siRNAs require Dcr-2 activity when they are produced from a synthetic miRNA-based backbone. In contrast, the mild suppression induced by the miRNA-like siRNA designs was not substantially dependent on Dcr-2.

## Discussion

In this paper we used a single PolII-based promoter to control the expression of small RNAs against CHIKV using the *D*. *melanogaster* pre-*miRNA-1* backbone as well as a fluorescent reporter (ZsYellow, ZsY) for the visualization of such expression. In *Ae*. *aegypti* and *Ae*. *albopictus* cells we tested the efficiency of small RNAs, fully complementary (shRNA-like) or with two mismatches (miRNA-like), to silence a CHIKV split replication system, and reporter constructs with targets in the CDS or 3’ UTR (Fig 1C). Finally, we investigated the importance of Dcr-2 in the silencing process.

Visualization of ZsY fluorescence (S3 Fig) could be used to indicate the presence/expression of the full transcript which includes the small RNAs and the ZsY reporter. Notably, ZsY expression was clear even in cells with a high degree of silencing for the CHIKV target RNAs suggesting any self-silencing of the ZsY reporter transcript was minimal, consistent with limited or no targeting of unspliced primary transcript. Previous studies investigating virus silencing by small RNAs used direct expression of small RNAs by selected promoters [5–7]. Detection of such expression depends on the quantification of the small RNAs, requiring the use of special extraction kits [5] and miSeq analysis [6]. When such studies are performed *in vivo*, dissection of the target tissues is necessary, adding an extra layer of complexity. The expression system described herein provides a useful tool, especially for *in vivo* work, potentially with cell-by-cell resolution. Expression levels of small RNAs may not precisely correlate with that of the reporter fluorophore, for example if processing or stability of these different components varies between cell types. However, visualization of the reporter fluorophore should provide a useful guide to identify transgenic mosquito lines with a suitable expression pattern of the full transcript encoding the small RNAs and associated reporter in relevant tissues, before the performance of, for example, time consuming infection studies.

Fully complementary small RNAs (shRNA-like siRNAs) were demonstrated to be significantly more efficient than those containing two mismatches (miRNA-like siRNAs) for silencing the three reporters CHIKVRep, CHILuc and LucCHI (Figs 2 and 3). This could be explained by the involvement of both Argonaute complexes (Ago1 and Ago2) by the shRNA-like siRNAs. The use of the pre-*miRNA-1* backbone was expected to result in a Dcr-1 dependent response [9,38] with the miRNA loaded into an Ago1-containing miRISC complex. Although we did not analyze the involvement of Ago pathways directly, our data are consistent with a model in which the miRNA-based designs were indeed processed by Dcr-1, as their activity was not affected by the absence of Dcr-2. The shRNA-like siRNAs lost much of their activity in Dcr-2-deficient cells, indicating processing primarily by Dcr-2. This suggests that the fully complementary small RNAs were sorted into the Ago2 pathway, as Dcr-2 and R2D2 are necessary to initiate the assembly of the small interference RNA-induced silencing complex (siRISC) [39–42]. The Ago1 (miRNA) and Ago2 (shRNA) pathways differ in their cleavage efficiency [32,33], which may explain the lower silencing effect of the miRNA-like siRNAs relative to equivalent shRNA-like siRNAs.

The poor efficiency of our miRNA-like siRNAs for silencing reporters, relative to the corresponding shRNA-like siRNAs, could additionally or alternatively be due to the selection of the target region within the nsP2 of CHIKV. miRNAs can be effective targeting the coding sequence but are often described as targeting 3’ UTR regions [43,44]. This, however, does not explain why the miRNA-like siRNAs were not more efficient than shRNA-like siRNA when using the LucCHI reporter which provided a 3’ UTR target.

The same small RNAs showed different ability to silence the three CHIKV reporters. The silencing was more efficient in CHILuc, followed by LucCHI and finally CHIKVRep, in *Ae*. *aegypti* and *Ae*. *albopictus* cell lines (Figs 2 and 3). This could be due to the influence of flanking RNA sequences affecting RNA folding and accessibility (S1 Fig). The differences in interference between CHILuc and LucCHI could also be related to the inherent characteristics of the small interference pathway involved, as fully complementary siRNAs usually target regions in the RNA to induce cleavage and degradation of the messenger RNA (mRNA), while the siRNAs that contain mismatches tend to identify targets complementary to their seed region (nucleotides 2–7 or 8) in the 3’ UTR repressing the translation of the mRNA into protein [32,43]. These reporters are, nevertheless, still useful to help eliminate small RNAs that will potentially be ineffective if tested with a split replication system (if available) or the virus. The lower silencing efficiency of the shRNA-like siRNAs against the CHIKVRep could be due to the characteristics of the split replication system itself. Here the shRNA-like siRNA is targeting the replication complex encoding mRNA expressed from plasmid pCHIKVRep2. Replication complex will in turn lead to expression of the NL located after the subgenomic promoter in pCHIKVRep1 (S4 Fig). Any replication complex that escaped the silencing machinery will then be capable of generating multiple copies of NL; the double-stranded replication intermediate itself is thought to be largely sheltered from the RNA interference (RNAi) system [45]. Inefficient silencing in this system may therefore allow substantial expression of the reporter. A similar difference in silencing efficiency between CHIKVRep and CHILuc was also observed in a mechanistically distinct silencing systems using Cas13b [13].

In general, suppression levels with shRNA-like siRNAs in *Ae*. *albopictus* cells (U4.4) were not as strong as in *Ae*. *aegypti* cells (Aag2) (compare Figs 2 and 3). This could be due to differences in promoter activity in the different cell lines. The relevance of this difference for viral silencing in cell culture and in mosquitoes is yet to be determined.

We provide here a direct comparison between siRNAs that are fully complementary or contain two mismatches against CHIKV reporters, and show that selected synthetic small RNAs that were fully complementary (shRNA-like) could more effectively reduce CHIKV replication with the split replication system, and more effectively suppress two different reporter constructs. It is clear that fully-complementary small RNAs expressed from an intron using a miRNA backbone generate an RNAi response; this system could potentially be used in mosquitoes to induce refractoriness against arboviruses. Small RNA expression to induce viral refractoriness has been shown previously against Zika, DENV and CHIKV [5,6,46], but these previous studies did not examine the effectiveness of their small RNA designs in the absence of Dcr-1 or Dcr-2 to attribute them to a pathway. It is possible that the authors were observing endo-siRNA based processing of perfect shRNAs mediated by Dcr-2 and Ago2. Considering that our fully complementary shRNA-like siRNAs were designed using a miRNA backbone, it would undoubtedly be interesting to investigate further the underlying mechanisms, for example whether the reduced expression of the reporters is due to (i) translation repression, in which case there would be little/no change to the amount of mRNA, or (ii) target degradation, in which case the reduction in protein (luciferase) expression would follow from a reduction in mRNA quantity. Both questions could be addressed, for example, by qRT-PCR or northern hybridization experiments.

Our design has the added benefit of allowing the visualization of the expression pattern of the parent mRNA transcript as a proxy for the resulting small RNAs, which will facilitate the development of transgenic mosquito lines suitable for viral challenge. Since the various components of our design (siRNA, intron, ZsY mRNA) are expressed from the same primary transcript, they are inevitably co-expressed. The visualization of ZsY is hence useful to identify lines that could be expressing the siRNAs in the correct location, which then can be further studied to quantify such expression in case subsequent processing/stability affects the degree of correlation between marker and siRNA.

In conclusion, the results obtained herein show that fully complementary small RNAs can be expressed from an intron when using a miRNA backbone, that they are highly efficient silencing different reporters, and are processed by the Dcr-2 pathway. The use of three different reporters, although cannot mimic an actual viral infection, facilitates the screening of numerous small RNAs for their efficiency before embarking in laborious experiments with virus, especially if those experiments require high containment levels. Our design, co-expressing a fluorophore, provides a tool that will be useful for the generation of transgenic mosquitoes, as it allows the visualization of the spatial and temporal expression of the small RNA, which is particularly useful when the transgenic mosquitoes are generated by random insertion and position effects are expected. The number of useful lines can then easily be narrowed down to those with the desired expression pattern, and then further investigated by more laborious techniques such as 21-mer sequencing and viral challenge.

## Supporting information

S1 Fig Target locations of small RNA 1–10 on the RNA secondary structures of the different reporters used predicted by MFold [16]. Most target regions have similar predicted structures in the different reporters. The highlighted sequences (purple) are the targets for the small RNAs.
(TIF)

S2 Fig **Predicted secondary stem-loop structure of small RNAs with MFold** [16] (A) Dmel microRNA *miR1*, (B) small RNA against firefly luciferase (FL) with mismatches in sequence (miRNA-like, miRT), (C) fully complementary small RNA against FL (shRNA-like, shRT), (D) small RNA 1 against target CHI of CHIKV with mismatches in sequence (miRNA-like, miR1), (E) fully complementary small RNA 1 against target CHI of CHIKV (shRNA-like, shR1). Essential mismatches in miRNA-like siRNAs as described in [9] are denoted by the black arrows. Bases highlighted in purple contain the target sequence.
(TIF)

S3 Fig Expression of ZsYellow (ZsY) reporter in cells transfected with selected small RNA constructs. Representative fluorescence microscope photos of (A-D) Aag2, (E-F) AF05, (I-L) Dcr-2 knockout AF319, (M-P) U4.4 and (Q-T) Dcr-2 deficient C6/36 cells. Acquisition parameters are listed in S18 Table. BF: Brightfield, NT: fully complementary non-targeting control small RNA, 6: fully complementary small RNA 6 targeting CHIKV nsP2.
(TIF)

S4 Fig Schematic of chikungunya virus (CHIKV) split replication assay with single small RNAs. A modified CHIKV genome is encoded by pCHIKVRep1 where the sequences of non-structural and structural proteins have been replaced with EGFP and nanoluciferase (NL), respectively. pCHIKVRep2 is co-transfected to supply the viral replicase polyprotein in *trans*. The targeted nsP2 region encodes an essential component of the CHIKV replication complex for the expression of NL that is under the control of the viral subgenomic promoter. The targeting ability of each small RNA tested can be measured from the change in NL expression levels. A firefly luciferase plasmid (FL) is co-transfected as a control for transfection efficiency.
(TIF)

S5 Fig Schematic of CHIKV viral reporter assays with single small RNAs. CHILuc and LucCHI contain the targeted 268 nt sequence from CHIKV encoded in the protein coding region and 3’UTR, respectively. Both reporters produce firefly luciferase (FL) and the targeting ability of each small RNA tested can be measured from the change in FL expression levels. A *Renilla* luciferase plasmid (RL) is co-transfected as a control for transfection efficiency.
(TIF)

S1 Table Panel of small RNAs designed for screening and primers used for cloning. miR: sequence with mismatches (small letters in bold), shR: fully complementary sequences. Target sequences are in capitals.
(DOCX)

S2 Table Results of statistical analyses performed for transfections with shRNA-like siRNAs and CHIKV split replication system in Aag2 cells. (DOCX)

S3 Table Results of statistical analyses performed for transfections with miRNA-like siRNAs and CHIKV split replication system in Aag2 cells. (DOCX)

S4 Table Results of statistical analyses performed for transfections with shRNA-like siRNAs and CHILuc in Aag2 cells. (DOCX)

S5 Table Results of statistical analyses performed for transfections with miRNA-like siRNAs and CHILuc in Aag2 cells. (DOCX)

S6 Table Results of statistical analyses performed for transfections with shRNA-like siRNAs and LucCHI in Aag2 cells. (DOCX)

S7 Table Results of statistical analyses performed for transfections with miRNA-like siRNAs and LucCHI in Aag2 cells. (DOCX)

S8 Table Results of statistical analyses performed for transfections with shRNA-like siRNAs and CHIKV split replication system in U4.4 cells. (DOCX)

S9 Table Results of statistical analyses performed for transfections with miRNA-like siRNAs and CHIKV split replication system in U4.4 cells. (DOCX)

S10 Table Results of statistical analyses performed for transfections with shRNA-like siRNAs and CHILuc in U4.4 cells. (DOCX)

S11 Table Results of statistical analyses performed for transfections with miRNA-like siRNAs and CHILuc in U4.4 cells. (DOCX)

S12 Table Results of statistical analyses performed for transfections with shRNA-like siRNAs and LucCHI in U4.4 cells. (DOCX)

S13 Table Results of statistical analyses performed for transfections with miRNA-like siRNAs and LucCHI in U4.4 cells. (DOCX)

S14 Table Results of statistical analyses performed for transfections with selected small RNAs and CHIKV split replication system, CHILuc and LucCHI in AF05 cells. (DOCX)

S15 Table Results of statistical analyses performed for transfections with selected small RNAs and CHIKV split replication system, CHILuc and LucCHI in AF319 cells. (DOCX)

S16 Table Results of statistical analyses performed for transfections with selected small RNAs and CHIKV split replication system, CHILuc and LucCHI in C6/36 cells. (DOCX)

S17 Table Plasmid mixtures for each experiment. (DOCX)

S18 Table Photo acquisition conditions of cells transfected with selected small RNA constructs. FOV: width of field of vision, Exp.: exposure time, FC: fluorochrome.
(DOCX)
