# Supplementary figures and images for: Intron-derived small RNAs for silencing viral RNAs in mosquito cells
Source: PLoS Negl Trop Dis. 2022 Jun 23;16(6):e0010548. doi: 10.1371/journal.pntd.0010548 (PMC9258879; doi:10.1371/journal.pntd.0010548)

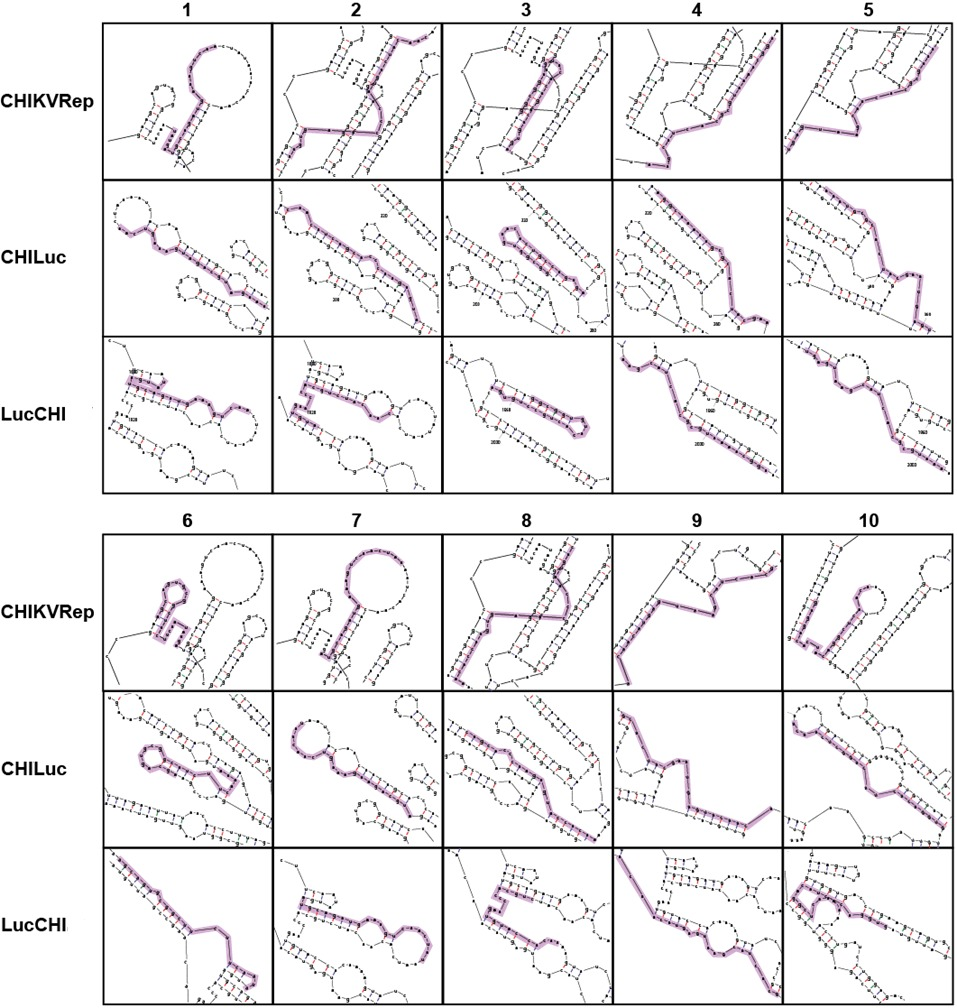

Supplement: S1 Fig — Most target regions have similar predicted structures in the different reporters. The highlighted sequences (purple) are the targets for the small RNAs. (TIF) [file pntd.0010548.s001.tif]

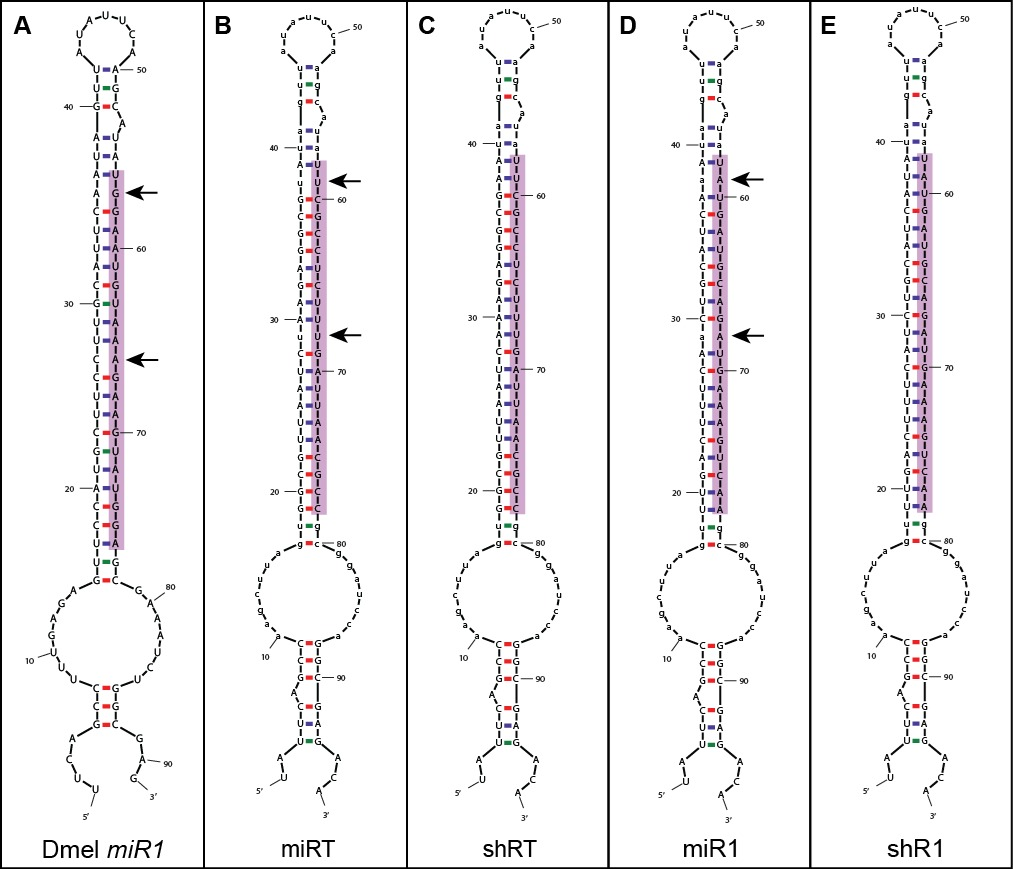

Supplement: S2 Fig — Predicted secondary stem-loop structure of small RNAs with MFold [16] (A) Dmel microRNA miR1, (B) small RNA against firefly luciferase (FL) with mismatches in sequence (miRNA-like, miRT), (C) fully complementary small RNA against FL (shRNA-like, shRT), (D) small RNA 1 against target CHI of CHIKV with mismatches in sequence (miRNA-like, miR1), (E) fully complementary small RNA 1 against target CHI of CHIKV (shRNA-like, shR1). Essential mismatches in miRNA-like siRNAs as described in [9] are denoted by the black arrows. Bases highlighted in purple contain the target sequence. (TIF) [file pntd.0010548.s002.tif]

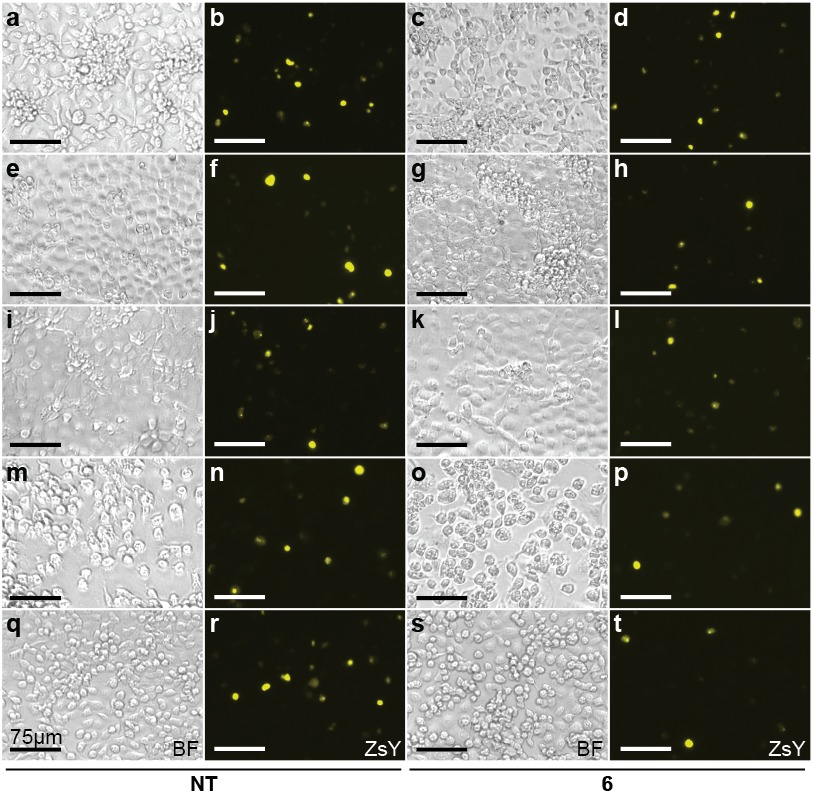

Supplement: S3 Fig — Representative fluorescence microscope photos of (A-D) Aag2, (E-F) AF05, (I-L) Dcr-2 knockout AF319, (M-P) U4.4 and (Q-T) Dcr-2 deficient C6/36 cells. Acquisition parameters are listed in S18 Table. BF: Brightfield, NT: fully complementary non-targeting control small RNA, 6: fully complementary small RNA 6 targeting CHIKV nsP2. (TIF) [file pntd.0010548.s003.tif]

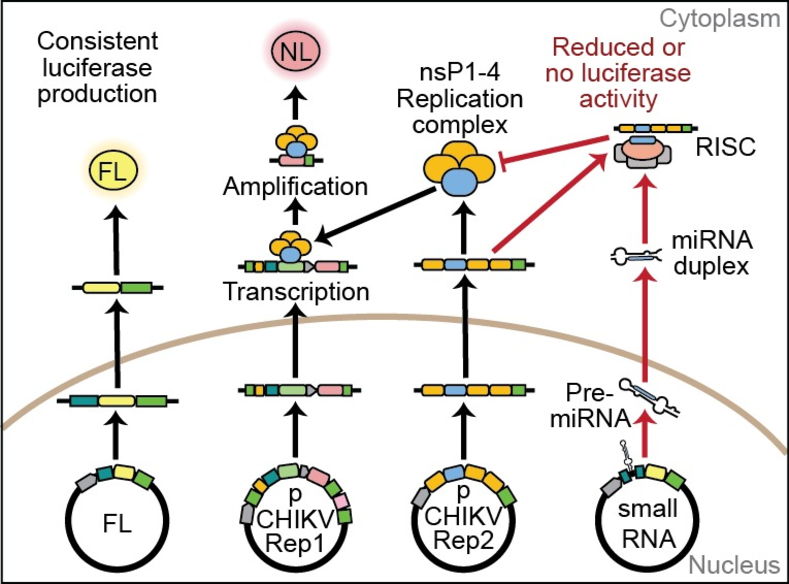

Supplement: S4 Fig — A modified CHIKV genome is encoded by pCHIKVRep1 where the sequences of non-structural and structural proteins have been replaced with EGFP and nanoluciferase (NL), respectively. pCHIKVRep2 is co-transfected to supply the viral replicase polyprotein in trans. The targeted nsP2 region encodes an essential component of the CHIKV replication complex for the expression of NL that is under the control of the viral subgenomic promoter. The targeting ability of each small RNA tested can be measured from the change in NL expression levels. A firefly luciferase plasmid (FL) is co-transfected as a control for transfection efficiency. (TIF) [file pntd.0010548.s004.tif]

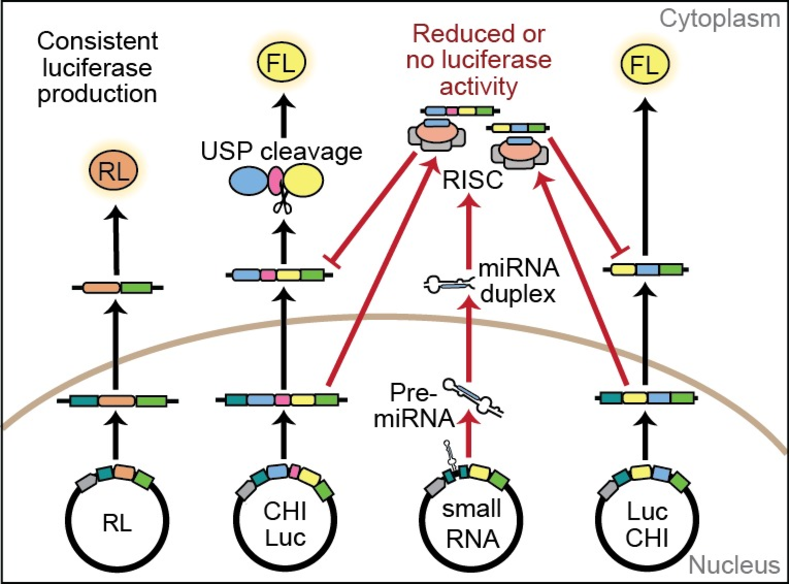

Supplement: S5 Fig — CHILuc and LucCHI contain the targeted 268 nt sequence from CHIKV encoded in the protein coding region and 3’UTR, respectively. Both reporters produce firefly luciferase (FL) and the targeting ability of each small RNA tested can be measured from the change in FL expression levels. A Renilla luciferase plasmid (RL) is co-transfected as a control for transfection efficiency. (TIF) [file pntd.0010548.s005.tif]
