# Supplementary material for: Intron-derived small RNAs for silencing viral RNAs in mosquito cells
Source: PLoS Negl Trop Dis. 2022 Jun 23;16(6):e0010548. doi: 10.1371/journal.pntd.0010548 (PMC9258879; doi:10.1371/journal.pntd.0010548)
Supplement: S1 Table — miR: sequence with mismatches (small letters in bold), shR: fully complementary sequences. Target sequences are in capitals. (DOCX) [file pntd.0010548.s006.docx]

S1Table. Panel of small RNAs designed for screening and primers used for cloning. miR: sequence with mismatches (small letters in bold), shR: fully complementary sequences. Target sequences are in capitals.

| **Small RNA** | **Forward Primer** | **Reverse Primer** |
| --- | --- | --- |
| miRNT | agcttagtTGGCCCACATcACCTCCGTcGtagttatattcaagcataCCACGGAGGTGATGTGGGCCAgcg | gatccgcTGGCCCACATCACCTCCGTGGtatgcttgaatataactaCgACGGAGGTgATGTGGGCCAacta |
| miRT | agcttagtGGCGTTAATCtAAGAGGCGtAtagttatattcaagcataTTCGCCTCTTTGATTAACGCCgcg | gatccgcGGCGTTAATCAAAGAGGCGAAtatgcttgaatataactaTaCGCCTCTTaGATTAACGCCacta |
| miR1 | agcttagtTTGACTTTCAaCTGCATCAaAtagttatattcaagcataTATGATGCAGATGAAAGTCAAgcg | gatccgcTTGACTTTCATCTGCATCATAtatgcttgaatataactaTtTGATGCAGtTGAAAGTCAAacta |
| miR2 | agcttagtTACACTTGGGaGCAGATGTaGtagttatattcaagcataCAACATCTGCACCCAAGTGTAgcg | gatccgcTACACTTGGGTGCAGATGTTGtatgcttgaatataactaCtACATCTGCtCCCAAGTGTAacta |
| miR3 | agcttagtTCACAGGCAGaGTACACCGgCtagttatattcaagcataGGCGGTGTACACTGCCTGTGAgcg | gatccgcTCACAGGCAGTGTACACCGCCtatgcttgaatataactaGcCGGTGTACtCTGCCTGTGAacta |
| miR4 | agcttagtTTCGTAGTGCcCATTTTGCgTtagttatattcaagcataAGGCAAAATGCGCACTACGAAgcg | gatccgcTTCGTAGTGCGCATTTTGCCTtatgcttgaatataactaAcGCAAAATGgGCACTACGAAacta |
| miR5 | agcttagtAAATGCGCACaACGAATGAcTtagttatattcaagcataACTCATTCGTAGTGCGCATTTgcg | gatccgcAAATGCGCACTACGAATGAGTtatgcttgaatataactaAgTCATTCGTtGTGCGCATTTacta |
| miR6 | agcttagtTATTGAAGAAcCCGCACTGgTtagttatattcaagcataAGCAGTGCGGCTTCTTCAATAgcg | gatccgcTATTGAAGAAGCCGCACTGCTtatgcttgaatataactaAcCAGTGCGGgTTCTTCAATAacta |
| miR7 | agcttagtATAGTTGACTaTCATCTGCtTtagttatattcaagcataATGCAGATGAAAGTCAACTATgcg | gatccgcATAGTTGACTTTCATCTGCATtatgcttgaatataactaAaGCAGATGAtAGTCAACTATacta |
| miR8 | agcttagtTTGTGGTACAgTTGGGTGCtGtagttatattcaagcataCTGCACCCAAGTGTACCACAAgcg | gatccgcTTGTGGTACACTTGGGTGCAGtatgcttgaatataactaCaGCACCCAAcTGTACCACAAacta |
| miR9 | agcttagtTGTTGTACTCtTTCGTAGTcCtagttatattcaagcataGCACTACGAATGAGTACAACAgcg | gatccgcTGTTGTACTCATTCGTAGTGCtatgcttgaatataactaGgACTACGAAaGAGTACAACAacta |
| miR10 | agcttagtTGTTGAGCCTcTAGTGTCCtCtagttatattcaagcataGTGGACACTACAGGCTCAACAgcg | gatccgcTGTTGAGCCTGTAGTGTCCACtatgcttgaatataactaGaGGACACTAgAGGCTCAACAacta |
| shRNT | agcttagtTGGCCCACATCACCTCCGTGGtagttatattcaagcataCCACGGAGGTGATGTGGGCCAgcg | gatccgcTGGCCCACATCACCTCCGTGGtatgcttgaatataactaCCACGGAGGTGATGTGGGCCAacta |
| shRT | agcttagtGGCGTTAATCAAAGAGGCGAAtagttatattcaagcataTTCGCCTCTTTGATTAACGCCgcg | gatccgcGGCGTTAATCAAAGAGGCGAAtatgcttgaatataactaTTCGCCTCTTTGATTAACGCCacta |
| shR1 | agcttagtTTGACTTTCATCTGCATCATAtagttatattcaagcataTATGATGCAGATGAAAGTCAAgcg | gatccgcTTGACTTTCATCTGCATCATAtatgcttgaatataactaTATGATGCAGATGAAAGTCAAacta |
| shR2 | agcttagtTACACTTGGGTGCAGATGTTGtagttatattcaagcataCAACATCTGCACCCAAGTGTAgcg | gatccgcTACACTTGGGTGCAGATGTTGtatgcttgaatataactaCAACATCTGCACCCAAGTGTAacta |
| shR3 | agcttagtTCACAGGCAGTGTACACCGCCtagttatattcaagcataGGCGGTGTACACTGCCTGTGAgcg | gatccgcTCACAGGCAGTGTACACCGCCtatgcttgaatataactaGGCGGTGTACACTGCCTGTGAacta |
| shR4 | agcttagtTTCGTAGTGCGCATTTTGCCTtagttatattcaagcataAGGCAAAATGCGCACTACGAAgcg | gatccgcTTCGTAGTGCGCATTTTGCCTtatgcttgaatataactaAGGCAAAATGCGCACTACGAAacta |
| shR5 | agcttagtAAATGCGCACTACGAATGAGTtagttatattcaagcataACTCATTCGTAGTGCGCATTTgcg | gatccgcAAATGCGCACTACGAATGAGTtatgcttgaatataactaACTCATTCGTAGTGCGCATTTacta |
| shR6 | agcttagtTATTGAAGAAGCCGCACTGCTtagttatattcaagcataAGCAGTGCGGCTTCTTCAATAgcg | gatccgcTATTGAAGAAGCCGCACTGCTtatgcttgaatataactaAGCAGTGCGGCTTCTTCAATAacta |
| shR7 | agcttagtATAGTTGACTTTCATCTGCATtagttatattcaagcataATGCAGATGAAAGTCAACTATgcg | gatccgcATAGTTGACTTTCATCTGCATtatgcttgaatataactaATGCAGATGAAAGTCAACTATacta |
| shR8 | agcttagtTTGTGGTACACTTGGGTGCAGtagttatattcaagcataCTGCACCCAAGTGTACCACAAgcg | gatccgcTTGTGGTACACTTGGGTGCAGtatgcttgaatataactaCTGCACCCAAGTGTACCACAAacta |
| shR9 | agcttagtTGTTGTACTCATTCGTAGTGCtagttatattcaagcataGCACTACGAATGAGTACAACAgcg | gatccgcTGTTGTACTCATTCGTAGTGCtatgcttgaatataactaGCACTACGAATGAGTACAACAacta |
| shR10 | agcttagtTGTTGAGCCTGTAGTGTCCACtagttatattcaagcataGTGGACACTACAGGCTCAACAgcg | gatccgcTGTTGAGCCTGTAGTGTCCACtatgcttgaatataactaGTGGACACTACAGGCTCAACAacta |
