# Supplementary material for: Intron-derived small RNAs for silencing viral RNAs in mosquito cells
Source: PLoS Negl Trop Dis. 2022 Jun 23;16(6):e0010548. doi: 10.1371/journal.pntd.0010548 (PMC9258879; doi:10.1371/journal.pntd.0010548)
Supplement: S2 Table — (DOCX) [file pntd.0010548.s007.docx]

S2 Table. Results of statistical analyses performed for transfections with shRNA-like siRNAs and CHIKV split replication system in Aag2 cells.

| Kruskal-Wallis rank sum test | | |  |  |  | |  |
| --- | --- | --- | --- | --- | --- | --- | --- |
| Kruskal-Wallis chi-squared = 187.36, df = 10, p-value < | | | | |  | 2.20E-16 | |
| Dunn's test | **Z** | **P.unadj** | **P.adj** |  |  | |  |
| sNT-s1 | 6.128367 | 8.88E-10 | 4.88E-09 |  |  | |  |
| sNT-s7 | 6.416316 | 1.4E-10 | 9.6E-10 |  |  | |  |
| sNT-s8 | 8.202181 | 2.36E-16 | 3.25E-15 |  |  | |  |
| sNT-s9 | 8.32725 | 8.27E-17 | 2.28E-15 |  |  | |  |
| sNT-s10 | 3.944027 | 8.01E-05 | 0.000192 |  |  | |  |
| sNT-s2 | 0.913292 | 0.361089 | 0.397198 |  |  | |  |
| sNT-s3 | -0.07271 | 0.942033 | 0.942033 |  |  | |  |
| sNT-s4 | 5.217983 | 1.81E-07 | 5.85E-07 |  |  | |  |
| sNT-s5 | 2.481014 | 0.013101 | 0.020587 |  |  | |  |
| sNT-s6 | 2.978381 | 0.002898 | 0.004981 |  |  | |  |
