# Supplementary material for: Intron-derived small RNAs for silencing viral RNAs in mosquito cells
Source: PLoS Negl Trop Dis. 2022 Jun 23;16(6):e0010548. doi: 10.1371/journal.pntd.0010548 (PMC9258879; doi:10.1371/journal.pntd.0010548)
Supplement: S3 Table — (DOCX) [file pntd.0010548.s008.docx]

S3 Table. Results of statistical analyses performed for transfections with miRNA-like siRNAs and CHIKV split replication system in Aag2 cells.

| Linear Mixed Model | | Differences were based on cuberoot transformed data. | | | |
| --- | --- | --- | --- | --- | --- |
| Random Effects | **Variance** | **Std.Dev.** |  |  |  |
| Experiment | 0.007543 | 0.08685 |  |  |  |
| Residual | 0.585168 | 0.76496 |  |  |  |
| Fixed Effects | **Estimate** | **Std. error** | **df** | **t value** | **Pr(>\|t\|)** |
| mNT-m1 | -0.16471 | 0.25499 | 185 | -0.646 | 0.519 |
| mNT-m7 | 0.09377 | 0.25499 | 185 | 0.368 | 0.713 |
| mNT-m8 | -0.25153 | 0.25499 | 185 | -0.986 | 0.325 |
| mNT-m9 | -0.01629 | 0.25499 | 185 | -0.064 | 0.949 |
| mNT-m10 | -0.24412 | 0.25499 | 185 | -0.957 | 0.34 |
| mNT-m2 | -0.04421 | 0.25499 | 185 | -0.173 | 0.863 |
| mNT-m3 | 0.26961 | 0.25499 | 185 | 1.057 | 0.292 |
| mNT-m4 | -0.35221 | 0.25499 | 185 | -1.381 | 0.169 |
| mNT-m5 | -0.39503 | 0.25499 | 185 | -1.549 | 0.123 |
| mNT-m6 | -0.3754 | 0.25499 | 185 | -1.472 | 0.143 |
