# Supplementary material for: Intron-derived small RNAs for silencing viral RNAs in mosquito cells
Source: PLoS Negl Trop Dis. 2022 Jun 23;16(6):e0010548. doi: 10.1371/journal.pntd.0010548 (PMC9258879; doi:10.1371/journal.pntd.0010548)
Supplement: S4 Table — (DOCX) [file pntd.0010548.s009.docx]

S4 Table. Results of statistical analyses performed for transfections with shRNA-like siRNAs and CHILuc in Aag2 cells.

| Kruskal-Wallis rank sum test | | |  |  |  |  |
| --- | --- | --- | --- | --- | --- | --- |
| Kruskal-Wallis chi-squared = 179.8, df = 11, p-value < | | | | |  | 2.20E-16 |
| Dunn's test | **Z** | **P.unadj** | **P.adj** |  |  |  |
| sNT-s1 | 5.997525 | 2E-09 | 1.32E-08 |  |  |  |
| sNT-s7 | 5.842854 | 5.13E-09 | 2.82E-08 |  |  |  |
| sNT-s8 | 9.06429 | 1.25E-19 | 8.28E-18 |  |  |  |
| sNT-s9 | 8.602942 | 7.77E-18 | 2.56E-16 |  |  |  |
| sNT-s10 | 4.205468 | 2.61E-05 | 8.19E-05 |  |  |  |
| sNT-s2 | 1.78139 | 0.074849 | 0.0988 |  |  |  |
| sNT-s3 | 2.245405 | 0.024742 | 0.039829 |  |  |  |
| sNT-s4 | 5.442841 | 5.24E-08 | 2.66E-07 |  |  |  |
| sNT-s5 | 3.909458 | 9.25E-05 | 0.000265 |  |  |  |
| sNT-s6 | 2.277406 | 0.022762 | 0.037557 |  |  |  |
| sNT-sT | 7.656245 | 1.91E-14 | 4.21E-13 |  |  |  |
