# Supplementary material for: Intron-derived small RNAs for silencing viral RNAs in mosquito cells
Source: PLoS Negl Trop Dis. 2022 Jun 23;16(6):e0010548. doi: 10.1371/journal.pntd.0010548 (PMC9258879; doi:10.1371/journal.pntd.0010548)
Supplement: S5 Table — (DOCX) [file pntd.0010548.s010.docx]

S5 Table. Results of statistical analyses performed for transfections with miRNA-like siRNAs and CHILuc in Aag2 cells.

| Linear Mixed Model | | Differences were based on squareroot transformed data. | | | |
| --- | --- | --- | --- | --- | --- |
| Random Effects | **Variance** | **Std.Dev.** |  |  |  |
| Experiment | 0.2118 | 0.4602 |  |  |  |
| Residual | 1.6462 | 1.283 |  |  |  |
| Fixed Effects | **Estimate** | **Std. error** | **df** | **t value** | **Pr(>\|t\|)** |
| mNT-m1 | -1.7962 | 0.4277 | 202 | -4.2 | 4.00E-05 |
| mNT-m7 | -0.5275 | 0.4277 | 202 | -1.233 | 0.21889 |
| mNT-m8 | -0.6934 | 0.4277 | 202 | -1.621 | 0.10651 |
| mNT-m9 | -1.8227 | 0.4277 | 202 | -4.262 | 3.11E-05 |
| mNT-m10 | -1.1467 | 0.4277 | 202 | -2.681 | 0.00794 |
| mNT-m2 | -1.5368 | 0.4277 | 202 | -3.593 | 0.00041 |
| mNT-m3 | -2.1158 | 0.4277 | 202 | -4.947 | 1.58E-06 |
| mNT-m4 | -2.1837 | 0.4277 | 202 | -5.106 | 7.59E-07 |
| mNT-m5 | -2.5568 | 0.4277 | 202 | -5.978 | 1.01E-08 |
| mNT-m6 | -0.2649 | 0.4277 | 202 | -0.619 | 0.53634 |
| mNT-mT | -4.5931 | 0.4277 | 202 | -10.74 | < 2e-16 |
