# Supplementary material for: Intron-derived small RNAs for silencing viral RNAs in mosquito cells
Source: PLoS Negl Trop Dis. 2022 Jun 23;16(6):e0010548. doi: 10.1371/journal.pntd.0010548 (PMC9258879; doi:10.1371/journal.pntd.0010548)
Supplement: S6 Table — (DOCX) [file pntd.0010548.s011.docx]

S6 Table. Results of statistical analyses performed for transfections with shRNA-like siRNAs and LucCHI in Aag2 cells.

| Kruskal-Wallis rank sum test | | |  |  |  |  |
| --- | --- | --- | --- | --- | --- | --- |
| Kruskal-Wallis chi-squared = 166.19, df = 11, p-value < | | | | |  | 2.20E-16 |
| Dunn's test | **Z** | **P.unadj** | **P.adj** |  |  |  |
| sNT-s1 | 5.658848 | 1.52E-08 | 1.01E-07 |  |  |  |
| sNT-s7 | 6.045527 | 1.49E-09 | 1.23E-08 |  |  |  |
| sNT-s8 | 8.053591 | 8.04E-16 | 2.65E-14 |  |  |  |
| sNT-s9 | 7.861585 | 3.79E-15 | 8.34E-14 |  |  |  |
| sNT-s10 | 3.402776 | 0.000667 | 0.001631 |  |  |  |
| sNT-s2 | 2.528081 | 0.011469 | 0.019919 |  |  |  |
| sNT-s3 | 2.528081 | 0.011469 | 0.020458 |  |  |  |
| sNT-s4 | 3.245437 | 0.001173 | 0.002497 |  |  |  |
| sNT-s5 | 3.253437 | 0.00114 | 0.002508 |  |  |  |
| sNT-s6 | 4.578813 | 4.68E-06 | 1.47E-05 |  |  |  |
| sNT-sT | 9.357633 | 8.15E-21 | 5.38E-19 |  |  |  |
