# Supplementary material for: Intron-derived small RNAs for silencing viral RNAs in mosquito cells
Source: PLoS Negl Trop Dis. 2022 Jun 23;16(6):e0010548. doi: 10.1371/journal.pntd.0010548 (PMC9258879; doi:10.1371/journal.pntd.0010548)
Supplement: S7 Table — (DOCX) [file pntd.0010548.s012.docx]

S7 Table. Results of statistical analyses performed for transfections with miRNA-like siRNAs and LucCHI in Aag2 cells.

| Kruskal-Wallis rank sum test | | |  |  |  |  |
| --- | --- | --- | --- | --- | --- | --- |
| Kruskal-Wallis chi-squared = 76.309, df = 11, p-value = | | | | |  | 7.60E-12 |
| Dunn's test | **Z** | **P.unadj** | **P.adj** |  |  |  |
| mNT-m1 | 3.810789 | 0.000139 | 0.000914 |  |  |  |
| mNT-m7 | 2.562749 | 0.010385 | 0.042837 |  |  |  |
| mNT-m8 | 2.186737 | 0.028762 | 0.090394 |  |  |  |
| mNT-m9 | 3.722786 | 0.000197 | 0.001182 |  |  |  |
| mNT-m10 | 1.594718 | 0.110775 | 0.215035 |  |  |  |
| mNT-m2 | 3.410776 | 0.000648 | 0.003054 |  |  |  |
| mNT-m3 | 3.216103 | 0.001299 | 0.005718 |  |  |  |
| mNT-m4 | 1.912061 | 0.055868 | 0.14182 |  |  |  |
| mNT-m5 | 1.296041 | 0.194961 | 0.329934 |  |  |  |
| mNT-m6 | 1.557383 | 0.11938 | 0.225116 |  |  |  |
| mNT-mT | 7.434905 | 1.05E-13 | 6.91E-12 |  |  |  |
