# Supplementary material for: Intron-derived small RNAs for silencing viral RNAs in mosquito cells
Source: PLoS Negl Trop Dis. 2022 Jun 23;16(6):e0010548. doi: 10.1371/journal.pntd.0010548 (PMC9258879; doi:10.1371/journal.pntd.0010548)
Supplement: S8 Table — (DOCX) [file pntd.0010548.s013.docx]

S8 Table. Results of statistical analyses performed for transfections with shRNA-like siRNAs and CHIKV split replication system in U4.4 cells.

| Linear Mixed Model | | Differences were based on squareroot transformed data. | | | |
| --- | --- | --- | --- | --- | --- |
| Random Effects | **Variance** | **Std.Dev.** |  |  |  |
| Experiment | 7.571 | 2.752 |  |  |  |
| Residual | 1.86 | 1.364 |  |  |  |
| Fixed Effects | **Estimate** | **Std. error** | **df** | **t value** | **Pr(>\|t\|)** |
| sNT-s1 | -6.5906 | 0.4546 | 185 | -14.498 | < 2e-16 |
| sNT-s7 | -9.297 | 0.4546 | 185 | -20.451 | < 2e-16 |
| sNT-s8 | -9.4852 | 0.4546 | 185 | -20.865 | < 2e-16 |
| sNT-s9 | -8.1199 | 0.4546 | 185 | -17.862 | < 2e-16 |
| sNT-s10 | -3.8246 | 0.4546 | 185 | -8.413 | 1.06E-14 |
| sNT-s2 | -2.6795 | 0.4546 | 185 | -5.894 | 1.75E-08 |
| sNT-s3 | -1.1947 | 0.4546 | 185 | -2.628 | 9.31E-03 |
| sNT-s4 | -4.5388 | 0.4546 | 185 | -9.984 | < 2e-16 |
| sNT-s5 | -3.18 | 0.4546 | 185 | -6.995 | 4.69E-11 |
| sNT-s6 | -5.8774 | 0.4546 | 185 | -12.929 | < 2e-16 |
