# Supplementary material for: Intron-derived small RNAs for silencing viral RNAs in mosquito cells
Source: PLoS Negl Trop Dis. 2022 Jun 23;16(6):e0010548. doi: 10.1371/journal.pntd.0010548 (PMC9258879; doi:10.1371/journal.pntd.0010548)
Supplement: S9 Table — (DOCX) [file pntd.0010548.s014.docx]

S9 Table. Results of statistical analyses performed for transfections with miRNA-like siRNAs and CHIKV split replication system in U4.4 cells.

| Linear Mixed Model | | Differences were based on log2 transformed data. | | | |
| --- | --- | --- | --- | --- | --- |
| Random Effects | **Variance** | **Std.Dev.** |  |  |  |
| Experiment | 0.09635 | 0.3104 |  |  |  |
| Residual | 0.08755 | 0.2959 |  |  |  |
| Fixed Effects | **Estimate** | **Std. error** | **df** | **t value** | **Pr(>\|t\|)** |
| mNT-m1 | 0.212416 | 0.098631 | 185 | 2.154 | 0.03256 |
| mNT-m7 | 0.079086 | 0.098631 | 185 | 0.802 | 0.42368 |
| mNT-m8 | -0.11051 | 0.098631 | 185 | -1.12 | 2.64E-01 |
| mNT-m9 | 0.126603 | 0.098631 | 185 | 1.284 | 0.20089 |
| mNT-m10 | 0.288217 | 0.098631 | 185 | 2.922 | 0.00391 |
| mNT-m2 | 0.116408 | 0.098631 | 185 | 1.18 | 2.39E-01 |
| mNT-m3 | -0.00801 | 0.098631 | 185 | -0.081 | 9.35E-01 |
| mNT-m4 | -0.04054 | 0.098631 | 185 | -0.411 | 6.82E-01 |
| mNT-m5 | 0.093805 | 0.098631 | 185 | 0.951 | 0.34281 |
| mNT-m6 | 0.02685 | 0.098631 | 185 | 0.272 | 0.78575 |
