# Supplementary material for: Intron-derived small RNAs for silencing viral RNAs in mosquito cells
Source: PLoS Negl Trop Dis. 2022 Jun 23;16(6):e0010548. doi: 10.1371/journal.pntd.0010548 (PMC9258879; doi:10.1371/journal.pntd.0010548)
Supplement: S10 Table — (DOCX) [file pntd.0010548.s015.docx]

S10 Table. Results of statistical analyses performed for transfections with shRNA-like siRNAs and CHILuc in U4.4 cells.

| Linear Mixed Model | | Differences were based on squareroot transformed data. | | | |
| --- | --- | --- | --- | --- | --- |
| Random Effects | **Variance** | **Std.Dev.** |  |  |  |
| Experiment | 0.09097 | 0.3016 |  |  |  |
| Residual | 0.05526 | 0.2351 |  |  |  |
| Fixed Effects | **Estimate** | **Std. error** | **df** | **t value** | **Pr(>\|t\|)** |
| sNT-s1 | -2.25266 | 0.07836 | 202 | -28.75 | < 2e-16 |
| sNT-s7 | -2.3097 | 0.07836 | 202 | -29.48 | < 2e-16 |
| sNT-s8 | -2.50433 | 0.07836 | 202 | -31.96 | < 2e-16 |
| sNT-s9 | -2.49558 | 0.07836 | 202 | -31.85 | < 2e-16 |
| sNT-s10 | -2.08412 | 0.07836 | 202 | -26.6 | < 2e-16 |
| sNT-s2 | -1.28644 | 0.07836 | 202 | -16.42 | < 2e-16 |
| sNT-s3 | -1.37923 | 0.07836 | 202 | -17.6 | < 2e-16 |
| sNT-s4 | -1.84794 | 0.07836 | 202 | -23.58 | < 2e-16 |
| sNT-s5 | -1.8002 | 0.07836 | 202 | -22.97 | < 2e-16 |
| sNT-s6 | -1.79122 | 0.07836 | 202 | -22.86 | < 2e-16 |
| sNT-sT | -2.4533 | 0.07836 | 202 | -31.31 | < 2e-16 |
