# Supplementary material for: Intron-derived small RNAs for silencing viral RNAs in mosquito cells
Source: PLoS Negl Trop Dis. 2022 Jun 23;16(6):e0010548. doi: 10.1371/journal.pntd.0010548 (PMC9258879; doi:10.1371/journal.pntd.0010548)
Supplement: S11 Table — (DOCX) [file pntd.0010548.s016.docx]

S11 Table. Results of statistical analyses performed for transfections with miRNA-like siRNAs and CHILuc in U4.4 cells.

| Linear Mixed Model | | Differences were based on squareroot transformed data. | | | |
| --- | --- | --- | --- | --- | --- |
| Random Effects | **Variance** | **Std.Dev.** |  |  |  |
| Experiment | 0.02982 | 0.1727 |  |  |  |
| Residual | 0.22534 | 0.4747 |  |  |  |
| Fixed Effects | **Estimate** | **Std. error** | **df** | **t value** | **Pr(>\|t\|)** |
| mNT-m1 | -0.7869 | 0.1582 | 202 | -4.973 | 1.41E-06 |
| mNT-m7 | -0.4018 | 0.1582 | 202 | -2.54 | 0.011853 |
| mNT-m8 | -0.3355 | 0.1582 | 202 | -2.12 | 0.035227 |
| mNT-m9 | -0.5755 | 0.1582 | 202 | -3.637 | 0.00035 |
| mNT-m10 | -0.2752 | 0.1582 | 202 | -1.739 | 0.083551 |
| mNT-m2 | -0.6065 | 0.1582 | 202 | -3.833 | 0.000169 |
| mNT-m3 | -0.9303 | 0.1582 | 202 | -5.879 | 1.68E-08 |
| mNT-m4 | -0.4311 | 0.1582 | 202 | -2.725 | 0.007 |
| mNT-m5 | -0.9897 | 0.1582 | 202 | -6.255 | 2.34E-09 |
| mNT-m6 | -0.3108 | 0.1582 | 202 | -1.964 | 0.050906 |
| mNT-mT | -2.0386 | 0.1582 | 202 | -12.884 | < 2e-16 |
