# Supplementary material for: Intron-derived small RNAs for silencing viral RNAs in mosquito cells
Source: PLoS Negl Trop Dis. 2022 Jun 23;16(6):e0010548. doi: 10.1371/journal.pntd.0010548 (PMC9258879; doi:10.1371/journal.pntd.0010548)
Supplement: S12 Table — (DOCX) [file pntd.0010548.s017.docx]

S12 Table. Results of statistical analyses performed for transfections with shRNA-like siRNAs and LucCHI in U4.4 cells.

| Kruskal-Wallis rank sum test | | |  |  |  |  |
| --- | --- | --- | --- | --- | --- | --- |
| Kruskal-Wallis chi-squared = 127.81, df = 11, p-value < | | | | |  | 2.20E-16 |
| Dunn's test | **Z** | **P.unadj** | **P.adj** |  |  |  |
| sNT-s1 | 3.640116 | 0.000273 | 0.000899 |  |  |  |
| sNT-s7 | 5.266835 | 1.39E-07 | 9.16E-07 |  |  |  |
| sNT-s8 | 5.650848 | 1.6E-08 | 1.51E-07 |  |  |  |
| sNT-s9 | 6.656213 | 2.81E-11 | 6.18E-10 |  |  |  |
| sNT-s10 | 2.60275 | 0.009248 | 0.018496 |  |  |  |
| sNT-s2 | 1.010699 | 0.312161 | 0.374593 |  |  |  |
| sNT-s3 | 1.789391 | 0.073552 | 0.115582 |  |  |  |
| sNT-s4 | 2.360076 | 0.018271 | 0.033497 |  |  |  |
| sNT-s5 | 2.344075 | 0.019074 | 0.034024 |  |  |  |
| sNT-s6 | 3.589448 | 0.000331 | 0.001041 |  |  |  |
| sNT-sT | 8.130927 | 4.26E-16 | 2.81E-14 |  |  |  |
