# Supplementary material for: Intron-derived small RNAs for silencing viral RNAs in mosquito cells
Source: PLoS Negl Trop Dis. 2022 Jun 23;16(6):e0010548. doi: 10.1371/journal.pntd.0010548 (PMC9258879; doi:10.1371/journal.pntd.0010548)
Supplement: S13 Table — (DOCX) [file pntd.0010548.s018.docx]

S13 Table. Results of statistical analyses performed for transfections with miRNA-like siRNAs and LucCHI in U4.4 cells.

| Kruskal-Wallis rank sum test | | |  |  |  |  |
| --- | --- | --- | --- | --- | --- | --- |
| Kruskal-Wallis chi-squared = 70.064, df = 11, p-value = | | | | |  | 1.19E-10 |
| Dunn's test | **Z** | **P.unadj** | **P.adj** |  |  |  |
| mNT-m1 | 2.008064 | 0.044636 | 0.1473 |  |  |  |
| mNT-m7 | 2.829424 | 0.004663 | 0.023675 |  |  |  |
| mNT-m8 | 1.717388 | 0.085908 | 0.226798 |  |  |  |
| mNT-m9 | 1.722722 | 0.084939 | 0.233582 |  |  |  |
| mNT-m10 | 0.392013 | 0.695049 | 0.790918 |  |  |  |
| mNT-m2 | 1.562717 | 0.118119 | 0.278424 |  |  |  |
| mNT-m3 | 1.061367 | 0.288523 | 0.464452 |  |  |  |
| mNT-m4 | -0.79203 | 0.428346 | 0.614583 |  |  |  |
| mNT-m5 | 0.552018 | 0.580936 | 0.737342 |  |  |  |
| mNT-m6 | 1.178704 | 0.238516 | 0.449773 |  |  |  |
| mNT-mT | 6.296201 | 3.05E-10 | 1.01E-08 |  |  |  |
