# Supplementary material for: Intron-derived small RNAs for silencing viral RNAs in mosquito cells
Source: PLoS Negl Trop Dis. 2022 Jun 23;16(6):e0010548. doi: 10.1371/journal.pntd.0010548 (PMC9258879; doi:10.1371/journal.pntd.0010548)
Supplement: S14 Table — (DOCX) [file pntd.0010548.s019.docx]

S14 Table. Results of statistical analyses performed for transfections with selected small RNAs and CHIKV split replication system, CHILuc and LucCHI in AF05 cells.

| AF05 | shRNA-like | CHIKVRep | |  |  |  |
| --- | --- | --- | --- | --- | --- | --- |
| Kruskal-Wallis rank sum test | | |  |  |  |  |
| Kruskal-Wallis chi-squared = 67.147, df = 4, p-value = | | | | |  | 9.08E-14 |
| Dunn's test | **Z** | **P.unadj** | **P.adj** |  |  |  |
| sNT-s7 | 5.08463 | 3.68E-07 | 1.23E-06 |  |  |  |
| sNT-s8 | 5.811917 | 6.18E-09 | 3.09E-08 |  |  |  |
| sNT-s9 | 7.298389 | 2.91E-13 | 2.91E-12 |  |  |  |
| sNT-s6 | 2.475328 | 0.013311 | 0.019016 |  |  |  |
|  |  |  |  |  |  |  |
| AF05 | **miRNA-like** | **CHIKVRep** | |  |  |  |
| Linear Mixed Model | | Differences were based on untransformed data. | | | | |
| Random Effects | **Variance** | **Std.Dev.** |  |  |  |  |
| Experiment | 28090 | 167.6 |  |  |  |  |
| Residual | 21718 | 147.4 |  |  |  |  |
| Fixed Effects | **Estimate** | **Std. error** | **df** | **t value** | **Pr(>\|t\|)** |  |
| mNT-m7 | 33.722 | 49.123 | 83 | 0.686 | 0.4943 |  |
| mNT-m8 | -91.556 | 49.123 | 83 | -1.864 | 0.0659 |  |
| mNT-m9 | -6.444 | 49.123 | 83 | -0.131 | 0.8959 |  |
| mNT-m6 | -143.833 | 49.123 | 83 | -2.928 | 0.0044 |  |
|  |  |  |  |  |  |  |
| AF05 | **shRNA-like** | **CHILuc** |  |  |  |  |
| Linear Mixed Model | | Differences were based on log2 transformed data. | | | | |
| Random Effects | **Variance** | **Std.Dev.** |  |  |  |  |
| Experiment | 6.935 | 2.633 |  |  |  |  |
| Residual | 3.455 | 1.859 |  |  |  |  |
| Fixed Effects | **Estimate** | **Std. error** | **df** | **t value** | **Pr(>\|t\|)** |  |
| sNT-s7 | -4.6929 | 0.6498 | 97 | -7.222 | 1.16E-10 |  |
| sNT-s8 | -6.2025 | 0.6498 | 97 | -9.545 | 1.29E-15 |  |
| sNT-s9 | -5.9625 | 0.6498 | 97 | -9.176 | 8.07E-15 |  |
| sNT-s6 | -3.3723 | 0.6498 | 97 | -5.19 | 1.16E-06 |  |
| sNT-sT | -6.2838 | 0.6498 | 97 | -9.67 | 6.9E-16 |  |

|  |  |  |  |  |  |  |
| --- | --- | --- | --- | --- | --- | --- |
| AF05 | **miRNA-like** | **CHILuc** |  |  |  |  |
| Kruskal-Wallis rank sum test | | |  |  |  |  |
| Kruskal-Wallis chi-squared = 32.903, df = 5, p-value = | | | | |  | 3.93E-06 |
| Dunn's test | **Z** | **P.unadj** | **P.adj** |  |  |  |
| mNT-m7 | 1.224766 | 0.220664 | 0.413744 |  |  |  |
| mNT-m8 | 1.514028 | 0.130019 | 0.278611 |  |  |  |
| mNT-m9 | 0.856613 | 0.391659 | 0.652765 |  |  |  |
| mNT-m6 | 1.529806 | 0.126065 | 0.315162 |  |  |  |
| mNT-mT | 5.153482 | 2.56E-07 | 3.84E-06 |  |  |  |
|  |  |  |  |  |  |  |
| AF05 | **shRNA-like** | **LucCHI** |  |  |  |  |
| One-way ANOVA | | Differences were based on squareroot transformed data. | | | | |
|  | **Df** | **Sum Sq** | **Mean Sq** | **F value** | **Pr(>F)** |  |
| smRNA | 5 | 305.5 | 61.1 | 24.83 | 2.74E-16 |  |
| Residuals | 102 | 251 | 2.46 |  |  |  |
| Tukey | **Estimate** | **Std. error** | **t value** | **Pr(>\|t\|)** |  |  |
| sNT-s7 | -3.64046 | 0.5229 | -6.962 | <0.001 |  |  |
| sNT-s8 | -4.33441 | 0.5229 | -8.289 | <0.001 |  |  |
| sNT-s9 | -4.42633 | 0.5229 | -8.465 | <0.001 |  |  |
| sNT-s6 | -2.67152 | 0.5229 | -5.109 | <0.001 |  |  |
| sNT-sT | -5.10466 | 0.5229 | -9.762 | <0.001 |  |  |
|  |  |  |  |  |  |  |
| AF05 | **miRNA-like** | **LucCHI** |  |  |  |  |
| Linear Mixed Model | | Differences were based on squareroot transformed data. | | | | |
| Random Effects | **Variance** | **Std.Dev.** |  |  |  |  |
| Experiment | 1.064 | 1.031 |  |  |  |  |
| Residual | 5.894 | 2.428 |  |  |  |  |
| Fixed Effects | **Estimate** | **Std. error** | **df** | **t value** | **Pr(>\|t\|)** |  |
| mNT-m7 | -2.9244 | 0.8213 | 99.012 | -3.561 | 0.00057 |  |
| mNT-m8 | -3.1217 | 0.8213 | 99.012 | -3.801 | 0.000249 |  |
| mNT-m9 | -0.7948 | 0.8213 | 99.012 | -0.968 | 0.335487 |  |
| mNT-m6 | -1.5027 | 0.8213 | 99.012 | -1.83 | 0.070305 |  |
| mNT-mT | -6.6513 | 0.8213 | 99.012 | -8.099 | 1.47E-12 |  |
