# Supplementary material for: Intron-derived small RNAs for silencing viral RNAs in mosquito cells
Source: PLoS Negl Trop Dis. 2022 Jun 23;16(6):e0010548. doi: 10.1371/journal.pntd.0010548 (PMC9258879; doi:10.1371/journal.pntd.0010548)
Supplement: S15 Table — (DOCX) [file pntd.0010548.s020.docx]

S15 Table. Results of statistical analyses performed for transfections with selected small RNAs and CHIKV split replication system, CHILuc and LucCHI in AF319 cells.

| AF319 | shRNA-like | CHIKVRep | |  |  |  |
| --- | --- | --- | --- | --- | --- | --- |
| Linear Mixed Model | | **Differences were based on squareroot transformed data.** | | | | |
| Random Effects | **Variance** | **Std.Dev.** |  |  |  |  |
| Experiment | 944.9 | 30.74 |  |  |  |  |
| Residual | 6966.1 | 83.46 |  |  |  |  |
| Fixed Effects | **Estimate** | **Std. error** | **df** | **t value** | **Pr(>\|t\|)** |  |
| sNT-s7 | 364.778 | 26.495 | 6.307 | 13.768 | 6.09E-06 |  |
| sNT-s8 | -33.722 | 27.821 | 83 | -1.212 | 0.229 |  |
| sNT-s9 | -45.722 | 27.821 | 83 | -1.643 | 0.104 |  |
| sNT-s6 | -134.694 | 27.821 | 83 | -4.841 | 5.89E-06 |  |
| sNT-sT | 5.278 | 27.821 | 83 | 0.19 | 0.85 |  |
|  |  |  |  |  |  |  |
| AF319 | **miRNA-like** | **CHIKVRep** | |  |  |  |
| Kruskal-Wallis rank sum test | | |  |  |  |  |
| Kruskal-Wallis chi-squared = 22.367, df = 4, p-value = | | | | |  | 0.000169 |
| Dunn's test | **Z** | **P.unadj** | **P.adj** |  |  |  |
| mNT-m7 | -4.20459 | 2.62E-05 | 0.000262 |  |  |  |
| mNT-m8 | -3.40068 | 0.000672 | 0.003361 |  |  |  |
| mNT-m9 | -2.96044 | 0.003072 | 0.01024 |  |  |  |
| mNT-m6 | -1.47703 | 0.139667 | 0.199525 |  |  |  |
|  |  |  |  |  |  |  |
| AF319 | **shRNA-like** | **CHILuc** |  |  |  |  |
| Kruskal-Wallis rank sum test | | |  |  |  |  |
| Kruskal-Wallis chi-squared = 59.053, df = 5, p-value = | | | | |  | 1.91E-11 |
| Dunn's test | **Z** | **P.unadj** | **P.adj** |  |  |  |
| sNT-s7 | 4.565639 | 4.98E-06 | 1.87E-05 |  |  |  |
| sNT-s8 | 5.635211 | 1.75E-08 | 2.62E-07 |  |  |  |
| sNT-s9 | 5.305293 | 1.12E-07 | 5.62E-07 |  |  |  |
| sNT-s6 | 1.415454 | 0.156935 | 0.261559 |  |  |  |
| sNT-sT | 5.58732 | 2.31E-08 | 1.73E-07 |  |  |  |

|  |  |  |  |  |  |  |
| --- | --- | --- | --- | --- | --- | --- |
| AF319 | **miRNA-like** | **CHILuc** |  |  |  |  |
| Linear Mixed Model | | Differences were based on squareroot transformed data. | | | | |
| Random Effects | **Variance** | **Std.Dev.** |  |  |  |  |
| Experiment | 1.662 | 1.289 |  |  |  |  |
| Residual | 1.269 | 1.127 |  |  |  |  |
| Fixed Effects | **Estimate** | **Std. error** | **df** | **t value** | **Pr(>\|t\|)** |  |
| mNT-m7 | -2.1722 | 0.3756 | 100 | -5.784 | 8.37E-08 |  |
| mNT-m8 | -2.5841 | 0.3756 | 100 | -6.881 | 5.27E-10 |  |
| mNT-m9 | -1.758 | 0.3756 | 100 | -4.681 | 8.98E-06 |  |
| mNT-m6 | -1.6458 | 0.3756 | 100 | -4.382 | 0.000029 |  |
| mNT-mT | -3.4017 | 0.3756 | 100 | -9.058 | 1.15E-14 |  |
|  |  |  |  |  |  |  |
| AF319 | **shRNA-like** | **LucCHI** |  |  |  |  |
| Kruskal-Wallis rank sum test | | |  |  |  |  |
| Kruskal-Wallis chi-squared = 35.763, df = 5, p-value = | | | | |  | 1.06E-06 |
| Dunn's test | **Z** | **P.unadj** | **P.adj** |  |  |  |
| sNT-s7 | 2.724484 | 0.00644 | 0.019321 |  |  |  |
| sNT-s8 | 2.66595 | 0.007677 | 0.016451 |  |  |  |
| sNT-s9 | 3.708916 | 0.000208 | 0.001041 |  |  |  |
| sNT-s6 | 1.261138 | 0.207259 | 0.259074 |  |  |  |
| sNT-sT | 5.443646 | 5.22E-08 | 7.83E-07 |  |  |  |
|  |  |  |  |  |  |  |
| AF319 | **miRNA-like** | **LucCHI** |  |  |  |  |
| Linear Mixed Model | | Differences were based on untransformed data. | | | | |
| Random Effects | **Variance** | **Std.Dev.** |  |  |  |  |
| Experiment | 16.24 | 4.03 |  |  |  |  |
| Residual | 259.86 | 16.12 |  |  |  |  |
| Fixed Effects | **Estimate** | **Std. error** | **df** | **t value** | **Pr(>\|t\|)** |  |
| mNT-m7 | -21.504 | 5.373 | 100 | -4.002 | 0.000121 |  |
| mNT-m8 | -20.226 | 5.373 | 100 | -3.764 | 0.000282 |  |
| mNT-m9 | -22.535 | 5.373 | 100 | -4.194 | 5.94E-05 |  |
| mNT-m6 | -23.95 | 5.373 | 100 | -4.457 | 2.17E-05 |  |
| mNT-mT | -44.713 | 5.373 | 100 | -8.321 | 4.61E-13 |  |
