# Supplementary material for: Intron-derived small RNAs for silencing viral RNAs in mosquito cells
Source: PLoS Negl Trop Dis. 2022 Jun 23;16(6):e0010548. doi: 10.1371/journal.pntd.0010548 (PMC9258879; doi:10.1371/journal.pntd.0010548)
Supplement: S16 Table — (DOCX) [file pntd.0010548.s021.docx]

S16 Table. Results of statistical analyses performed for transfections with selected small RNAs and CHIKV split replication system, CHILuc and LucCHI in C6/36 cells.

| C6/36 | shRNA-like | CHIKVRep | |  |  |  |
| --- | --- | --- | --- | --- | --- | --- |
| Linear Mixed Model | | Differences were based on untransformed data. | | | | |
| Random Effects | **Variance** | **Std.Dev.** |  |  |  |  |
| Experiment | 5523 | 74.32 |  |  |  |  |
| Residual | 12023 | 109.65 |  |  |  |  |
| Fixed Effects | **Estimate** | **Std. error** | **df** | **t value** | **Pr(>\|t\|)** |  |
| sNT-s7 | -69.611 | 36.55 | 83 | -1.905 | 0.060302 |  |
| sNT-s8 | 33.556 | 36.55 | 83 | 0.918 | 0.361238 |  |
| sNT-s9 | -82.333 | 36.55 | 83 | -2.253 | 2.69E-02 |  |
| sNT-s6 | 44.889 | 36.55 | 83 | 1.228 | 2.23E-01 |  |
|  |  |  |  |  |  |  |
| C6/36 | **miRNA-like** | **CHIKVRep** | |  |  |  |
| Linear Mixed Model | | Differences were based on log2 transformed data. | | | | |
| Random Effects | **Variance** | **Std.Dev.** |  |  |  |  |
| Experiment | 0.002729 | 0.05224 |  |  |  |  |
| Residual | 0.086735 | 0.29451 |  |  |  |  |
| Fixed Effects | **Estimate** | **Std. error** | **df** | **t value** | **Pr(>\|t\|)** |  |
| mNT-m7 | -0.22608 | 0.09817 | 83 | -2.303 | 0.0238 |  |
| mNT-m8 | -0.14254 | 0.09817 | 83 | -1.452 | 0.1503 |  |
| mNT-m9 | -0.18127 | 0.09817 | 83 | -1.847 | 0.0684 |  |
| mNT-m6 | -0.13465 | 0.09817 | 83 | -1.372 | 0.1739 |  |
|  |  |  |  |  |  |  |
| C6/36 | **shRNA-like** | **CHILuc** |  |  |  |  |
| Linear Mixed Model | | Differences were based on log2 transformed data. | | | | |
| Random Effects | **Variance** | **Std.Dev.** |  |  |  |  |
| Experiment | 0.1028 | 0.3206 |  |  |  |  |
| Residual | 1.3543 | 1.1637 |  |  |  |  |
| Fixed Effects | **Estimate** | **Std. error** | **df** | **t value** | **Pr(>\|t\|)** |  |
| sNT-s7 | -2.1598 | 0.3879 | 100 | -5.568 | 2.18E-07 |  |
| sNT-s8 | -1.4274 | 0.3879 | 100 | -3.68 | 0.000378 |  |
| sNT-s9 | -1.9467 | 0.3879 | 100 | -5.018 | 2.27E-06 |  |
| sNT-s6 | -1.357 | 0.3879 | 100 | -3.498 | 0.000701 |  |
| sNT-sT | -1.439 | 0.3879 | 100 | -3.71 | 0.000341 |  |

|  |  |  |  |  |  |  |
| --- | --- | --- | --- | --- | --- | --- |
| C6/36 | **miRNA-like** | **CHILuc** |  |  |  |  |
| Kruskal-Wallis rank sum test | | |  |  |  |  |
| Kruskal-Wallis chi-squared = 33.942, df = 5, p-value = | | | | |  | 2.45E-06 |
| Dunn's test | **Z** | **P.unadj** | **P.adj** |  |  |  |
| mNT-m7 | 3.485423 | 0.000491 | 0.002457 |  |  |  |
| mNT-m8 | 3.144863 | 0.001662 | 0.006231 |  |  |  |
| mNT-m9 | 2.884121 | 0.003925 | 0.011775 |  |  |  |
| mNT-m6 | 4.017549 | 5.88E-05 | 0.000441 |  |  |  |
| mNT-mT | 5.624569 | 1.86E-08 | 2.79E-07 |  |  |  |
|  |  |  |  |  |  |  |
| C6/36 | **shRNA-like** | **LucCHI** |  |  |  |  |
| Linear Mixed Model | | Differences were based on squareroot transformed data. | | | | |
| Random Effects | **Variance** | **Std.Dev.** |  |  |  |  |
| Experiment | 0.001311 | 0.03621 |  |  |  |  |
| Residual | 0.261409 | 0.51128 |  |  |  |  |
| Fixed Effects | **Estimate** | **Std. error** | **df** | **t value** | **Pr(>\|t\|)** |  |
| sNT-s7 | -1.3991 | 0.1704 | 100 | -8.209 | 8.05E-13 |  |
| sNT-s8 | -0.9048 | 0.1704 | 100 | -5.309 | 6.66E-07 |  |
| sNT-s9 | -1.5962 | 0.1704 | 100 | -9.366 | 2.43E-15 |  |
| sNT-s6 | -0.8856 | 0.1704 | 100 | -5.196 | 1.08E-06 |  |
| sNT-sT | -1.0471 | 0.1704 | 100 | -6.144 | 1.65E-08 |  |
|  |  |  |  |  |  |  |
| C6/36 | **miRNA-like** | **LucCHI** |  |  |  |  |
| Linear Mixed Model | | Differences were based on untransformed data. | | | | |
| Random Effects | **Variance** | **Std.Dev.** |  |  |  |  |
| Experiment | 2.054 | 1.433 |  |  |  |  |
| Residual | 5.308 | 2.304 |  |  |  |  |
| Fixed Effects | **Estimate** | **Std. error** | **df** | **t value** | **Pr(>\|t\|)** |  |
| mNT-m7 | -3.6791 | 0.7679 | 100 | -4.791 | 5.78E-06 |  |
| mNT-m8 | -4.3123 | 0.7679 | 100 | -5.615 | 1.76E-07 |  |
| mNT-m9 | -5.7043 | 0.7679 | 100 | -7.428 | 3.77E-11 |  |
| mNT-m6 | -4.9088 | 0.7679 | 100 | -6.392 | 5.25E-09 |  |
| mNT-mT | -7.1497 | 0.7679 | 100 | -9.31 | 3.22E-15 |  |
