# Supplementary material for: Intron-derived small RNAs for silencing viral RNAs in mosquito cells
Source: PLoS Negl Trop Dis. 2022 Jun 23;16(6):e0010548. doi: 10.1371/journal.pntd.0010548 (PMC9258879; doi:10.1371/journal.pntd.0010548)
Supplement: S17 Table — (DOCX) [file pntd.0010548.s022.docx]

S17 Table. Plasmid mixtures for each experiment.

| Small RNAs with CHIKV split replication system | |
| --- | --- |
| Reporter | 10ng pCHIKVRep1, 1ng pCHIKVRep2 |
| Small RNA | 20ng of miRNA-like or shRNA-like |
| Transfection control | 0.2ng pHr5Fluc |
| Small RNAs with synthetic reporter CHILuc | |
| Reporter | 2ng AGG1221:pCHIKVLuc |
| Small RNA | 20ng of miRNA-like or shRNA-like |
| Transfection control | 50ng pRL-Opie2 |
| Small RNAs with synthetic reporter LucCHI | |
| Reporter | 2ng AGG1220:pLucCHIKV |
| Small RNA | 20ng of miRNA-like or shRNA-like |
| Transfection control | 50ng pRL-Opie2 |
