# Supplementary material for: Intron-derived small RNAs for silencing viral RNAs in mosquito cells
Source: PLoS Negl Trop Dis. 2022 Jun 23;16(6):e0010548. doi: 10.1371/journal.pntd.0010548 (PMC9258879; doi:10.1371/journal.pntd.0010548)
Supplement: S18 Table — FOV: width of field of vision, Exp.: exposure time, FC: fluorochrome. (DOCX) [file pntd.0010548.s023.docx]

S18 Table. Photo acquisition conditions of cells transfected with selected small RNA constructs. FOV: width of field of vision, Exp.: exposure time, FC: fluorochrome.

| Figure | Conditions | FOV (mm) | Exp. (ms) | Gain | FC |
| --- | --- | --- | --- | --- | --- |
| Supplementary Figure 3. a, c, e, g, i, k, m, o, q, s | BF | 0.87 | 1 | 10 | NA |
| Supplementary Figure 3. b, d, f, h, j, l, n, p, r, t | GFP | 0.87 | 7.5 | 7.5 | ZsYellow, Excitation nm: 529, Emission nm: 539 |
